# Comparative analysis of differential gene expression indicates divergence in ontogenetic strategies of leaves in two conifer genera

**DOI:** 10.1002/ece3.8611

**Published:** 2022-02-16

**Authors:** Cynthia Webster, Laura Figueroa‐Corona, Iván David Méndez‐González, Lluvia Álvarez‐Soto, David B. Neale, Juan Pablo Jaramillo‐Correa, Jill L. Wegrzyn, Alejandra Vázquez‐Lobo

**Affiliations:** ^1^ 7712 Department of Ecology and Evolutionary Biology University of Connecticut Storrs Connecticut USA; ^2^ 7180 Departamento de Ecología Evolutiva Instituto de Ecología Universidad Nacional Autónoma de México Ciudad de México Mexico; ^3^ 6614 Department of Biological Sciences University of Pittsburgh Pittsburgh Pennsylvania USA; ^4^ 27783 Facultad de Ciencias Biológicas Universidad Autónoma del Estado de Morelos Cuernavaca México; ^5^ Department of Plant Sciences University of California Davis California USA; ^6^ 27783 Centro de Investigación en Biodiversidad y Conservación Universidad Autónoma del Estado de Morelos Cuernavaca México

**Keywords:** cell wall and cutin biosynthesis, conifer leaf development, Heteroblasty, *Juniperus flaccida*, *Pinus cembroides*, RNA‐seq analysis

## Abstract

In land plants, heteroblasty broadly refers to a drastic change in morphology during growth through ontogeny. *Juniperus flaccida* and *Pinus cembroides* are conifers of independent lineages known to exhibit leaf heteroblasty between the juvenile and adult life stage of development. Juvenile leaves of *P. cembroides* develop spirally on the main stem and appear decurrent, flattened, and needle‐like; whereas adult photosynthetic leaves are triangular or semi‐circular needle‐like, and grow in whorls on secondary or tertiary compact dwarf shoots. By comparison, *J. flaccida* juvenile leaves are decurrent and needle‐like, and adult leaves are compact, short, and scale‐like. Comparative analyses were performed to evaluate differences in anatomy and gene expression patterns between developmental phases in both species. RNA from 12 samples was sequenced and analyzed with available software. They were assembled de novo from the RNA‐Seq reads. Following assembly, 63,741 high‐quality transcripts were functionally annotated in *P. cembroides* and 69,448 in *J. flaccida*. Evaluation of the orthologous groups yielded 4140 shared gene families among the four references (adult and juvenile from each species). Activities related to cell division and development were more abundant in juveniles than adults in *P. cembroides*, and more abundant in adults than juveniles in *J. flaccida*. Overall, there were 509 up‐regulated and 81 down‐regulated genes in the juvenile condition of *P. cembroides* and 14 up‐regulated and 22 down‐regulated genes in *J. flaccida*. Gene interaction network analysis showed evidence of co‐expression and co‐localization of up‐regulated genes involved in cell wall and cuticle formation, development, and phenylpropanoid pathway, in juvenile *P. cembroides* leaves. Whereas in *J. flaccida*, differential expression and gene interaction patterns were detected in genes involved in photosynthesis and chloroplast biogenesis. Although *J. flaccida* and *P. cembroides* both exhibit leaf heteroblastic development, little overlap was detected, and unique genes and pathways were highlighted in this study.

## Graphical abstract

*Juniperus flaccida* and *Pinus cembroides* exhibit leaf heteroblasty between the juvenile and adult life stage of development. Comparative analyses were performed to evaluate differences in anatomy and gene expression patterns between developmental phases in both species.

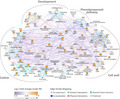

## 1 INTRODUCTION

Leaves of land plants display gradual changes in shape and size during growth, nonetheless some species undergo drastic developmental changes in morpho‐anatomy through ontogeny. Plant species that present these abrupt developmental changes were called heteroblastic for the first time by Goebel (1889), who distinguished them from homoblastic plants with gradual changes in shape and anatomy of their leaves and branches (Zotz et al., 2011). In recent decades, the term heteroblasty has been used more broadly to refer, as well, to gradual and/or small changes during development and aging (Allsopp, 1967; Greenwood et al., 2009), and it involves both morpho‐anatomical, physiological and biochemical characters (Jones, 1999). Regardless of whether the change is gradual or abrupt, there is a recognition that heteroblasty is genetically regulated, therefore, although the timing and morphology of heteroblastic transitions could be influenced by environmental signals, ontogenetic changes are not driven by the environment (e.g., Climent et al., 2006; Ehmig et al., 2019; Gamage & Jesson, 2007; Ostria‐Gallardo et al., 2016). Heteroblasty has been described in species of herbaceous (Burns, 2005; Guzmán & Antonio, 2015; Jones & Watson, 2001) and woody plants (Jaya et al., 2010; Lester, 1968; Rose et al., 2019), in different lineages of the phylogeny of land plants, and in different environmental conditions (Allsopp, 1967; Gamage & Jesson, 2007; Jones, 1999). Due to its convergent evolution, this feature is generally assumed to be an adaptation to the different biotic and abiotic environmental conditions surrounding juvenile and adult plants (Ehmig et al., 2019; Zotz et al., 2011), such as light and water availability (Day, 1998; James & Bell, 2000; Rose et al., 2019) or herbivory (Clark & Burns, 2015).

**FIGURE 1:**
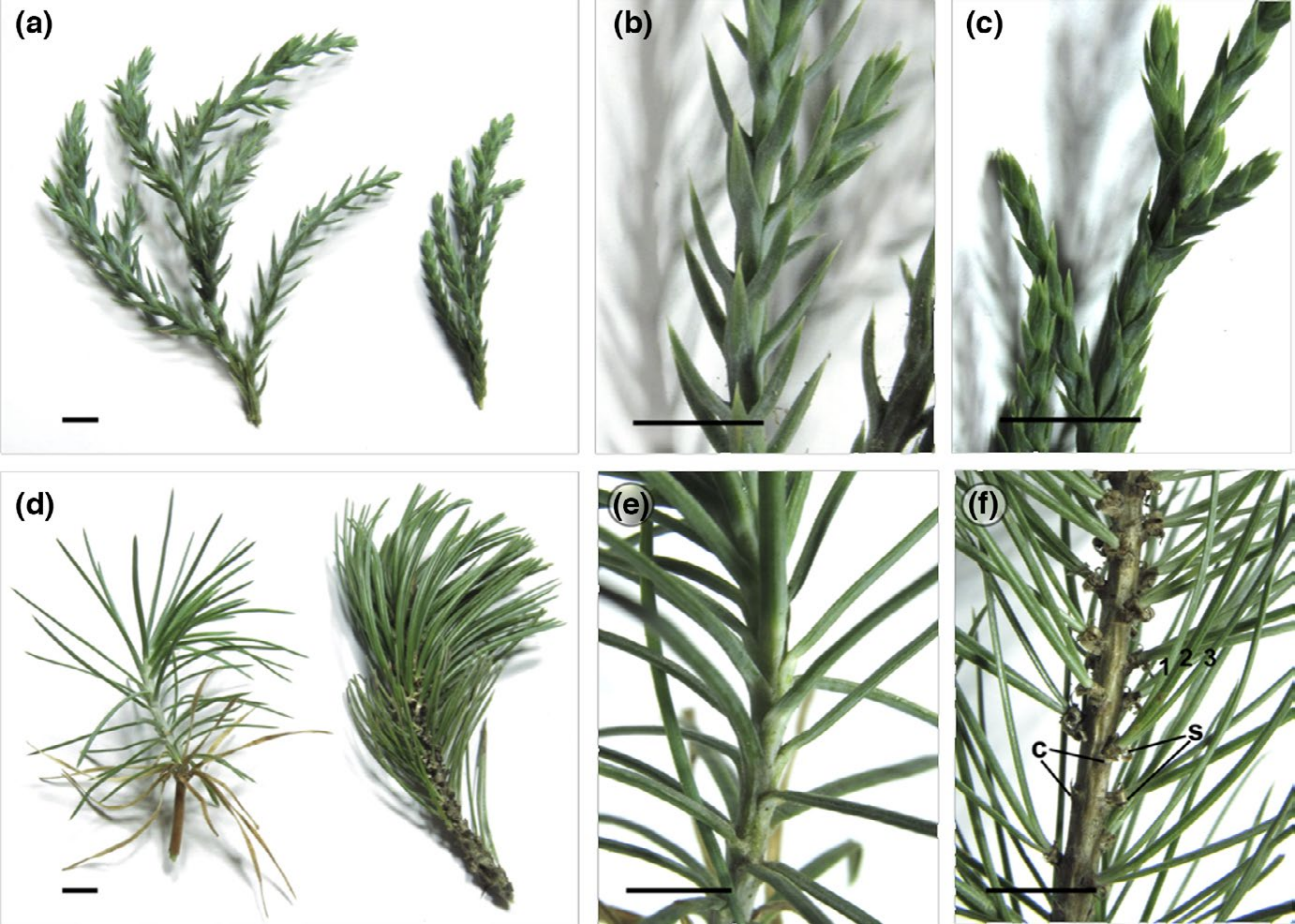
Comparisons of leaf arrangements in juvenile and adult branches of *J. flaccida* and *P. cembroides*. *J. flaccida* shoots (a–c). General view of juvenile and adult shoots (a). Closer view of juvenile (b) and adult shoots (c). *P. cembroides* shoots (d–f). General view of juvenile and adult shoots (d). Closer view of juvenile (e) and adult shoots (f) with fascicles of three needles. s, sheath; c, cataphyll. Black bars, 1 cm

**FIGURE 2:**
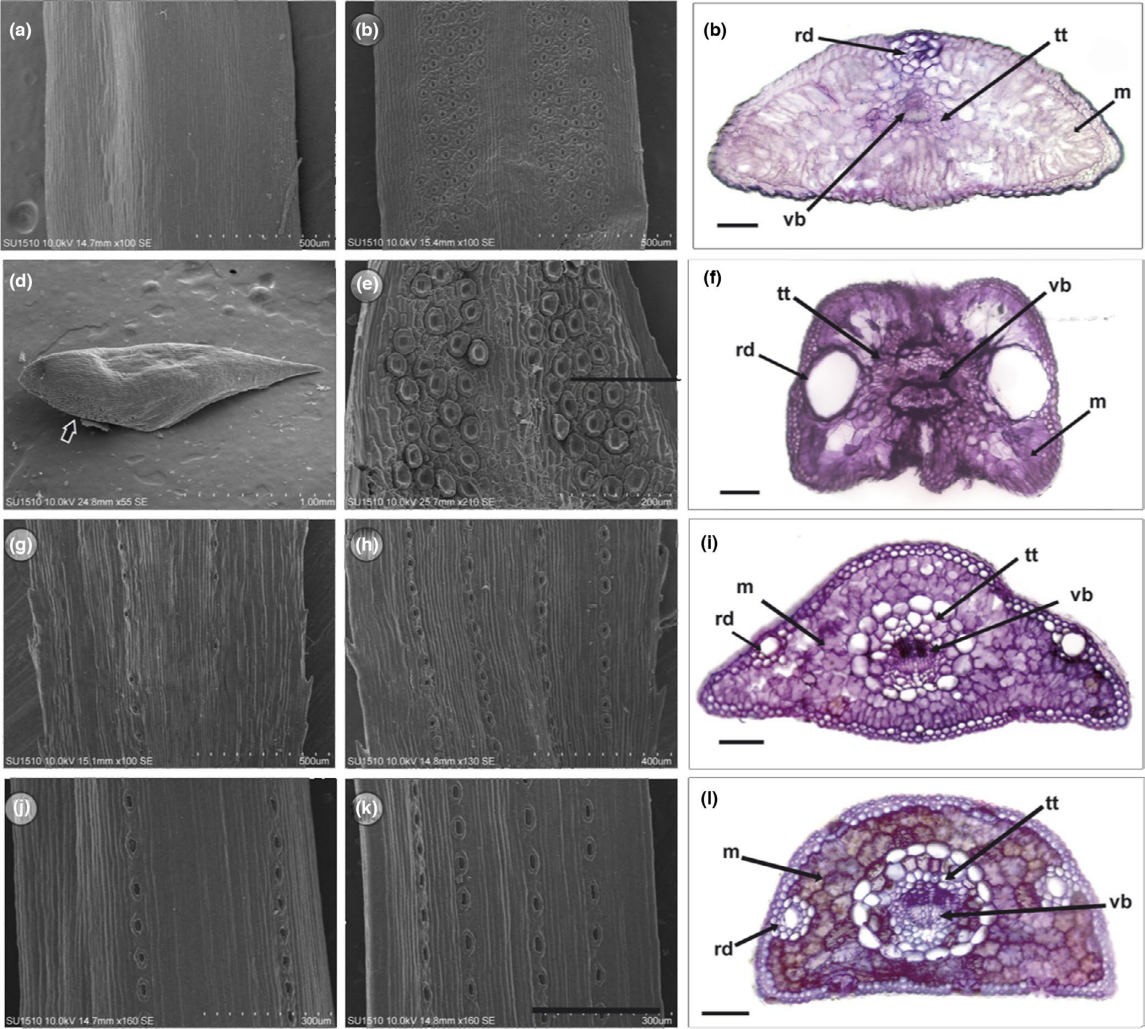
Anatomical comparisons in juvenile and adult leaves of *J. flaccida* and *P. cembroides*. Anatomical observations of juvenile and adult leaves of *J. flaccida* (a–f). Scanning electron microscopy observations (SEM) of the abaxial (a) and adaxial (b) sides, and a cross‐section of juvenile leaves (c). SEM of the abaxial (d) and adaxial (e) sides, and a cross‐section of two adult scales and stem (f). Anatomical observations of juvenile and adult leaves of *P. cembroides* (g–l). SEM of the abaxial (g) and adaxial (h) sides, and a cross‐section of juvenile needles (i). SEM of the abaxial (j) and adaxial (k) sides, and a cross‐section of adult needles (l). m, mesophyll; rd, resin duct; tt, transfusion tissue; vb, vascular bundle. Black bars, 100 μM

In contrast to the convergent evolution reported in angiosperms, a marked ancestral heteroblasty is present in two independent coniferous lineages within the Cupressaceae and Pinaceae families. On one hand, occurrence of juvenile leaves is a common character for species of Cupressaceae *s*.*s*. (Little, 2006), a clade integrated by the Callitroideae and Cupressoideae subfamilies (Gadek et al., 2000; Yang et al., 2012). Species in this clade exhibit juvenile and adult shoots with similar developmental patterns but bearing distinctive leaves, while the juveniles are decurrent short needle‐like, the adult ones are compact short scale‐like leaves (Bartel, 1993; Eckenwalder, 2009; e.g., Figure 1). Special attention has been paid to the morphology of juvenile leaves in Cupressaceae *s*.*s*., since some species exhibit neoteny and retain juvenile leaves throughout their life (Adams, 2014; Adams et al., 2013; Farjon et al., 2002). On the other hand, noticeable heteroblasty is well documented in the *Pinus* genus, where observed changes are not only in the leaf morphology but strikingly in shoot developmental patterns (Farjon & Styles, 1997; Lanner, 1976; Lester, 1968; Perry, 1991). In pines, juvenile leaves are decurrent, flattened, needle‐like, developing spirally on both the main stem and primary branches, in contrast to adult photosynthetic leaves, which are triangular or semi‐circular needle‐like, growing in whorls on secondary or tertiary compact dwarf shoots (fascicles; e.g., Figures 1 and 2). The extent of heteroblasty in other genera of the Pinaceae is not clear, since juvenile vegetative phases are not well described, likely the result of ephemeral juvenile stages or poorly distinguished transitions.

Species of Pinaceae and Cupressaceae are often dominant life forms in forests of temperate regions, nevertheless, the two most diverse genera of conifers (*Pinus* and *Juniperus*) have also spread to arid and semi‐arid environments of tropical regions (Gernandt & Vazquez‐Lobo, 2014). Evolution of heteroblasty likely precedes the diversification of both lineages during the Mesozoic. Therefore, this ontogenetic character in conifers is not necessarily an adaptation to current habitat conditions. Nonetheless, physiological studies have shown advantages for the establishment of young individuals in semi‐arid environments in both Cupressaceae *s*.*s*. (Miller et al., 1995; Tanaka‐Oda et al., 2010) and *Pinus* (Climent et al., 2006). Evidence of heterochronic differences in the transition from juvenile to adult leaves (Climent et al., 2011; Jones, 1999; Lester, 1968) and on intraspecific plasticity in timing of this transition influenced by the environment (Climent et al., 2006; Lloret & Granzow‐de la Cerda, 2013) suggests that different ontogenetic strategies have evolved in conifer species. Moreover, development of juvenile‐like shoots in mature trees after branch damage, or in rapidly growing shoots, is common in junipers (Adams, 2019; Bartel, 1993). In *Pinus*, development of juvenile‐like shoots after juvenile–adult phase transition is infrequent, but it can take place. For example, occurrence of epicormic juvenile‐like shoots has been reported in *P. canariensis* and *P. rigida* after fire damage (Climent et al., 2004; Gucker, 2007), after trimming in young trees of *P. echinata* (Bormann, 1955), and in mature trees of *P. maximartinezii* (Moreno‐Letelier and Vázquez‐Lobo, personal observation). Therefore, the incidence of juvenile‐like shoots in the adult vegetative phase of conifers indicates that this developmental pattern in some cases could be activated under environmental stress.

The genetic basis of heteroblasty have been studied mainly in angiosperm herbaceous species (Huijser & Schmid, 2011), characterized by gradual changes of both leaf shape and size during juvenile to adult transitions (Zotz et al., 2011). Changes between juvenile and adult leaves, such as the presence of trichomes and serration, were initially associated with the expression of two microRNAs (miRNAs) in *Arabidopsis thaliana* and *Zea mays* (Lauter et al., 2007; Wu & Scott Poethig, 2006). In juvenile leaves, *miR156* represses expression of *SQUAMOSA PROMOTER BINDING PROTEIN*‐*like* (*SPL*) genes, which promote vegetative phase change and flowering (Usami et al., 2009; Wu & Scott Poethig, 2006). Expression of *miR156* decreases with plant age, whereas expression of both *SPL* genes and *miR172* transcripts increases (Huijser & Schmid, 2011). An identified target for *miR172* is an *APETALA2*‐*like* gene, a floral repressor implicated in juvenile leaf traits (Lauter et al., 2007). In short‐lived plant species, the transition from juvenile to adult phase could occur simultaneously with the transition to reproductive phase, even though, similar changes in *miR172*, *miR156*, and *SPL* expression patterns have been documented in angiosperm trees and semi‐woody species, where morphological changes are notorious and transitions to adult leaves begin earlier than the vegetative phase change (Huijser & Schmid, 2011; Silva et al., 2019; Wang et al., 2011). A more detailed characterization of the genes involved in tree heteroblasty was carried out by means of transcriptomic and differential expression analyses (DEs) in the temperate tree species *Gevuina avellana* (Proteaceae) (Ostria‐Gallardo et al., 2016), identifying in addition to the *SPL* genes, developmental genes of the *MADS*‐*box* family, and genes involved in auxin synthesis and jasmonate activity, expressing differentially through the juvenile–adult transition.

Considering that most conifer species reach the reproductive phase several years after the transition to adult vegetative phase and that partial reversions to juvenile shoots may occur under environmental signals, it is possible to infer that heteroblasty in conifers is uncoupled with the transition to the reproductive phase. This creates an optimal system to study vegetative phase changes in plants (Huijser & Schmid, 2011). Physiological studies on *Pinus* and *Juniperus* have shown functional differences between juvenile and adult leaves (Bormann, 1955; Miller et al., 1995; Wright, 1970), though, there is no information regarding the genetic basis of these differences and other functional metabolic changes have not been characterized. The dimensions of adult trees, the long generation and reproductive cycle times, as well as the challenges associated with conifer genomes, have delayed the development of experimental and analytical approaches for this group of seed plants. Nevertheless, it is now possible to characterize functional changes through comparisons of gene expression patterns through RNA‐seq methods, which can detect differential expression of underlying genes and associated metabolic pathways (Marguerat & Bähler, 2010; Marioni et al., 2008), without prior knowledge of the specific sequences of the taxa under study. Therefore, we set out to characterize the differences in both anatomy and gene expression patterns between juvenile and adult leaves of two species of heteroblastic conifers (*P. cembroides* and *J. flaccida*) that can be found in sympatry in the semi‐arid zones of Mexico (Figure S1). We assess gene differential expression within the framework of the morpho‐anatomical and physiological differences that have been reported for the two types of leaves in junipers and pines.

## 2 RESULTS

### 2.1 Morpho‐anatomical differences between juvenile and adult leaves

Juvenile and adult shoots of *J. flaccida* bear leaves growing alternately along stems in a similar way (Figure 1a), although the juvenile stages of *J. flaccida* are distinguished by presenting a greater internodal distance and needle‐like leaves (Figure 1b), whereas the adult stages are characterized by compact scale‐like leaves (Figure 1c). In *P. cembroides*, juvenile shoots (Figure 1d) have flattened needle‐like leaves growing helically along a primary branch (Figure 1e), whereas in adult stages, needles develop in secondary or tertiary dwarf shoots (fascicles) integrated by two or three needles, grouped by a basal sheath developing axillary to a cataphyll (Figure 1f).

Microscopy observations show that juvenile leaves of *J. flaccida* have non‐serrated margins, no stomata on the abaxial surface (Figure 2a), and two longitudinal clusters of stomata along almost the entire leaf on the adaxial surface (Figure 2b). Internally, juvenile leaves bear one dorsal resin duct surrounded by large hypodermal cells, abundant mesophyll, and a central vascular bundle surrounded by compact transfusion tissue (Figure 2c). Epidermal and hypodermal cells are square shaped with thick cell walls, arranged in a single layer (Figure 2c). In adult scales of *J. flaccida* (Figure 2d‐f), a few stomata were found on the basal area of the abaxial face (Figure 2d), while the adaxial face is almost completely occupied by two disordered clusters of stomata and leaf margins incipiently serrated (Figure 2e). The cross‐sections of the adult leaves of *J. flaccida* include the stem, since the conductive tissue (xylem and phloem) does not penetrate the leaves; in turn, abundant transfusion tissue connects the vascular bundle with mesophyll (Figure 2f). Epidermal cells are small and compact, with thick cell walls, while hypodermis is absent. The dorsal resin duct occupies a considerable area of the leaves and is surrounded by compact epidermal cells (Figure 2f).

Juvenile leaves of *P. cembroides* have serrated margins (Figure 2g,h), two lines of stomata on the abaxial side (Figure 2g), and four on the adaxial side (Figure 2h). Pine juvenile leaves have two dorsal resin ducts under the epidermis surrounded by one layer of hypodermal cells, and a central vascular bundle surrounded by abundant transfusion tissue (Figure 2i). Epidermal and hypodermal cells are rounded with thick walls and arranged in single layers (Figure 2i). Adult needles of *P. cembroides* have non‐serrated margins, and similarly to juvenile needles, two rows of stomata on the abaxial side and four on the adaxial side (Figure 2j,k). In contrast with juvenile leaves, the resin ducts lie below the hypodermis and are surrounded by at least two layers of hypodermic cells (Figure 2l).

### 2.2 Transcriptome Assembly of *J. flaccida* and *P. cembroides*

*Juniperus flaccida* and *P. cembroides* were each composed of six paired‐end libraries, three juvenile and three adult. Following quality control, the total reads of adult juniper (JF1A, JF2A, JF3A) ranged between 56,134,644 and 63,763,344, while those of juvenile juniper (JF1J, JF2J, JF3J) ranged between 55,875,578 and 68,276,504. Comparatively, adult pine (PC1A, PC2A, PC3A) reads ranged between 37,255,402 and 47,498,316 and those of juvenile pine (PC1J, PC2J, PC3J) ranged between 42,030,740 and 51,844,340 (File S1).

Upon combining the independent assemblies of 12 libraries from Trinity, a total of 461,071 transcripts in *P. cembroides* and 577,348 in *J. flaccida* were present. This number was reduced to 231,054 transcripts (N50: 759bp) in *P. cembroides* and 229,054 transcripts (N50:717bp) in *J. flaccida* following frame selection with TransDecoder, that served to identify and retain long open reading frames (ORFs) with homology to known proteins. A total of 2074 transcripts were flagged as contaminants and removed from the *J. flaccida* assembly and 2050 from *P. cembroides* in the first round of EnTAP (Eukaryotic Non‐Model Transcriptome Annotation Pipeline). After clustering and removing sequences less than 300 bp, a second round resulted in the removal of an additional 83 transcripts in *J. flaccida* and 98 in *P. cembroides*. Combined, both conifers were associated with a diverse group of species; but the vast majority of them were bacterial: *Acinetobacter baumannii*, *Escherichia coli*, *Soehngenia saccharolytica*, and *Klebsiella pneumoniae*. After eliminating all identified contaminants, *P. cembroides* and *J. flaccida* were left with 63,741 and 69,448 transcripts, respectively (Table 1; Table S1). Overall, the single BUSCO (Benchmarking Universal Single‐Copy Orthologs) completeness for *J. flaccida* was 36.3% and for *P. cembroides* 30.1%. Lower scores were expected, given the samples were only collected from a single tissue type (needle).

**TABLE 1:** Summary of de novo assembled, frame selected, and final reference transcriptomes for both juniper and pine

Sample	Total transcripts	Total transcripts >500 bp	Average length (bp)	Transcript N50 (bp)
De novo assemblies—Trinity (FASTA)				
JF1A	159,283	66,774	588.661	516
JF2A	78,364	28,349	538.043	2039
JF3A	84,729	16,336	428.552	348
JF1J	123,905	35,549	478.675	366
JF2J	20,308	3907	427.049	322
JF3J	110,759	26,496	452.666	1015
PC1A	68,352	25,958	553.586	1222
PC2A	63,913	19,694	492.163	433
PC3A	65,228	23,396	525.753	319
PC1J	77,776	27,419	521.422	471
PC2J	103,756	35,550	513.244	797
PC3J	82,046	27,185	509.079	1112
Frame selected—TransDecoder (CDS)				
Juniper combined	229,054	78,234	511.222	717
Pine combined	231,054	82,265	511.606	759
Final clustered transcriptome—VSEARCH (CDS)				
Juniper combined	69,448	21,167	502.07	492
Pine combined	63,741	19,344	491.22	1089

The total transcripts with a high‐quality sequence similarity search alignment (NCBI Plant RefSeq) were 24,172 (34.8%) in *J. flaccida* and 23,247 (36.4%) in *P. cembroides* with primary annotations from *Amborella trichopoda*, *Cucurbita pepo*, and *Arabidopsis thaliana*. The total unique sequences annotated in *J. flaccida* and *P. cembroides*—including the gene family and/or sequence similarity search (NCBI RefSeq, TAIR [The Arabidopsis Information Resource], PLAZA, and EggNOG)—were 67.1% and 72.4%, respectively (Table S2).

### 2.3 Differential expression analysis

**FIGURE 3:**
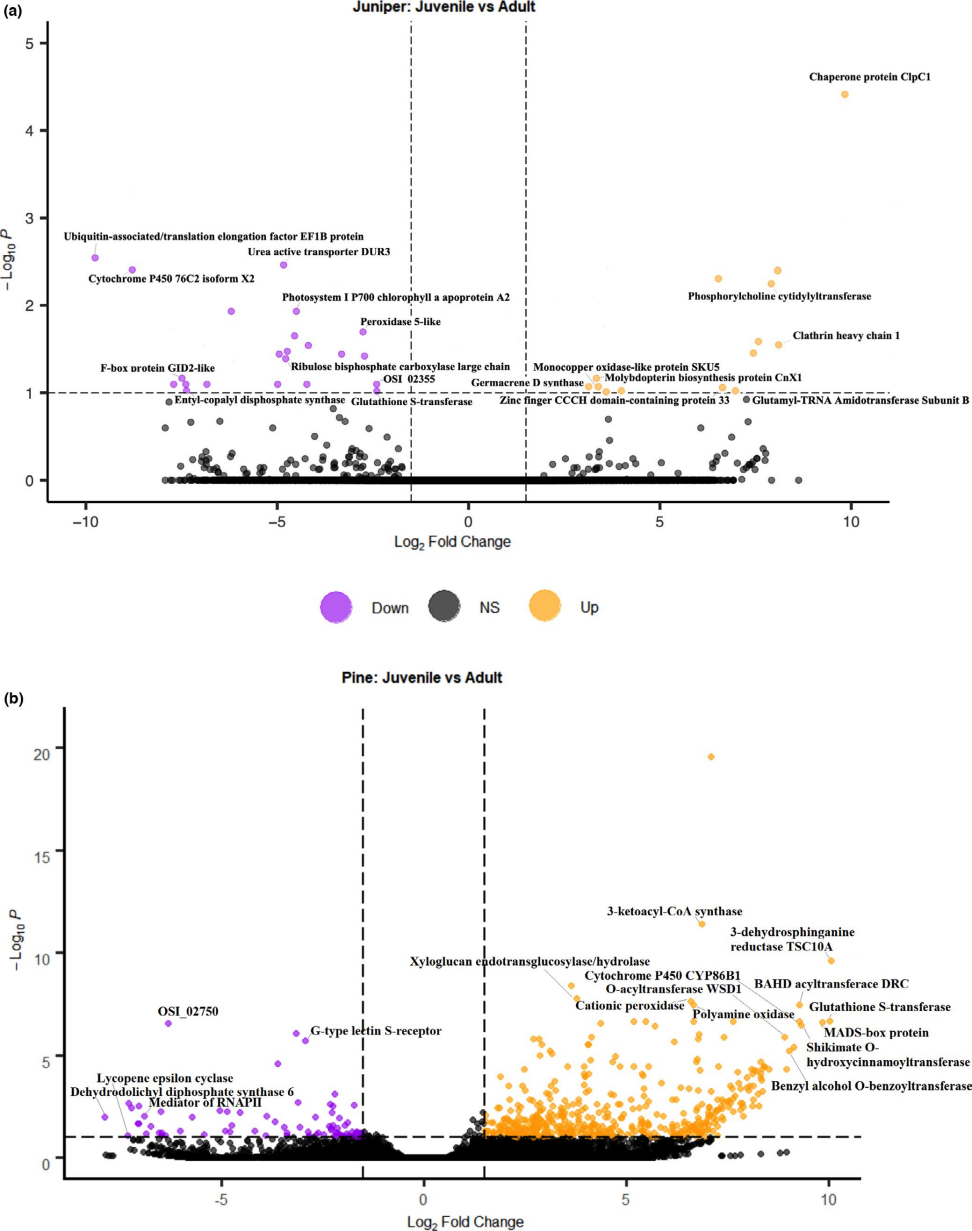
Volcano plot for differentially expressed genes of *J. flaccida* and *P. cembroides*. Significant up‐regulated (orange) and down‐regulated (purple) differentially expressed genes of both species within bounds (dashed line) of log2 fold change ≥1.5 (*x*‐axis) and ‐log10*p* ≥ 1 (*y*‐axis), with all non‐significant genes shown in black (a–b). A total of 14 up‐regulated and 22 down‐regulated genes shown in *J. flaccida* (a) and 509 up‐regulated and 81 down‐regulated genes in *P. cembroides* (b), some with labeled annotations

The six libraries pseudoaligned via Kallisto to the assembled transcriptome of *P. cembroides* had mapping rates ranging from 47.9% to 54.2%, while the six libraries pseudoaligned to *J. flaccida* ranged from 24.8% to 56.3% (Table S3). Lower alignment rates are attributed to contaminant removal, frame selection, and the more sensitive approach implemented by Kallisto for transcriptome mappings. Prior to frame selection, alignment rates were higher, ranging from 64.8% to 73.6% in *P. cembroides* and 58.7% to 75.0% in *J. flaccida* (Table S4). While contaminants were filtered from the assembled transcriptomes, they were not pre‐filtered at the read level. Kraken2 analysis revealed classification rates ranging from 21.01% to 33.93% in *P. cembroides* and 22.88% to 69.63% in *J. flaccida* (Table S5). Given classified reads cannot map to the reference genome in most cases, a lower mapping rate is expected. Preliminary examination of expression profiles by age class did not reveal a clear pattern (via principal component analysis [PCA]) but this is expected when sampling natural populations (Shahid Shaukat et al., 2016) (Figure S2). A total of 36 *J. flaccida* transcripts were differentially expressed, 14 up‐regulated (juvenile expressed) and 22 down‐regulated (adult expressed) (Figure 3a). Meanwhile, there were 590 *P. cembroides* differentially expressed (DE) genes, 509 up‐regulated and 81 down‐regulated (Figure 3b). When further partitioning log2 fold change by increments of 1.5–2, 2–4, and 4+, 8% of *P. cembroides* genes (File S2) and none of the *J. flaccida* genes (File S3) had a log2 fold change between 1.5 and 2, while 43% of *P. cembroides* genes and 28% of *J. flaccida* genes had a log2 fold change between 2 and 4, and 49% of *P. cembroides* genes and 72% of *J. flaccida* genes had greater than a four‐fold level of differential expression. In the *J. flaccida* juvenile gene set, chaperone protein ClpC1 was the most up‐regulated (9.82 fold change); this gene is thought to play a role in photosystem I biogenesis, chloroplast function, and leaf development (Sjögren et al., 2004). In the adult samples, EF1B (−9.76), a ubiquitin‐associated/translation elongation factor protein, was significantly down‐regulated. In juvenile *P. cembroides*, 3‐dehydrosphinganine reductase TSC10A (10.28), needed for sphingolipid biosynthesis (Chao et al., 2011), and glutathione S‐transferase GST5 (10.05), were the most significant up‐regulated genes. Among those with a functional annotation in adult *P. cembroides*, dehydrodolichyl diphosphate synthase 6 (−7.89) was strongly down‐regulated along with a mediator of RNA polymerase II transcription, MED20A (−7.04).

### 2.4 Functional classification of GO terms

**FIGURE 4:**
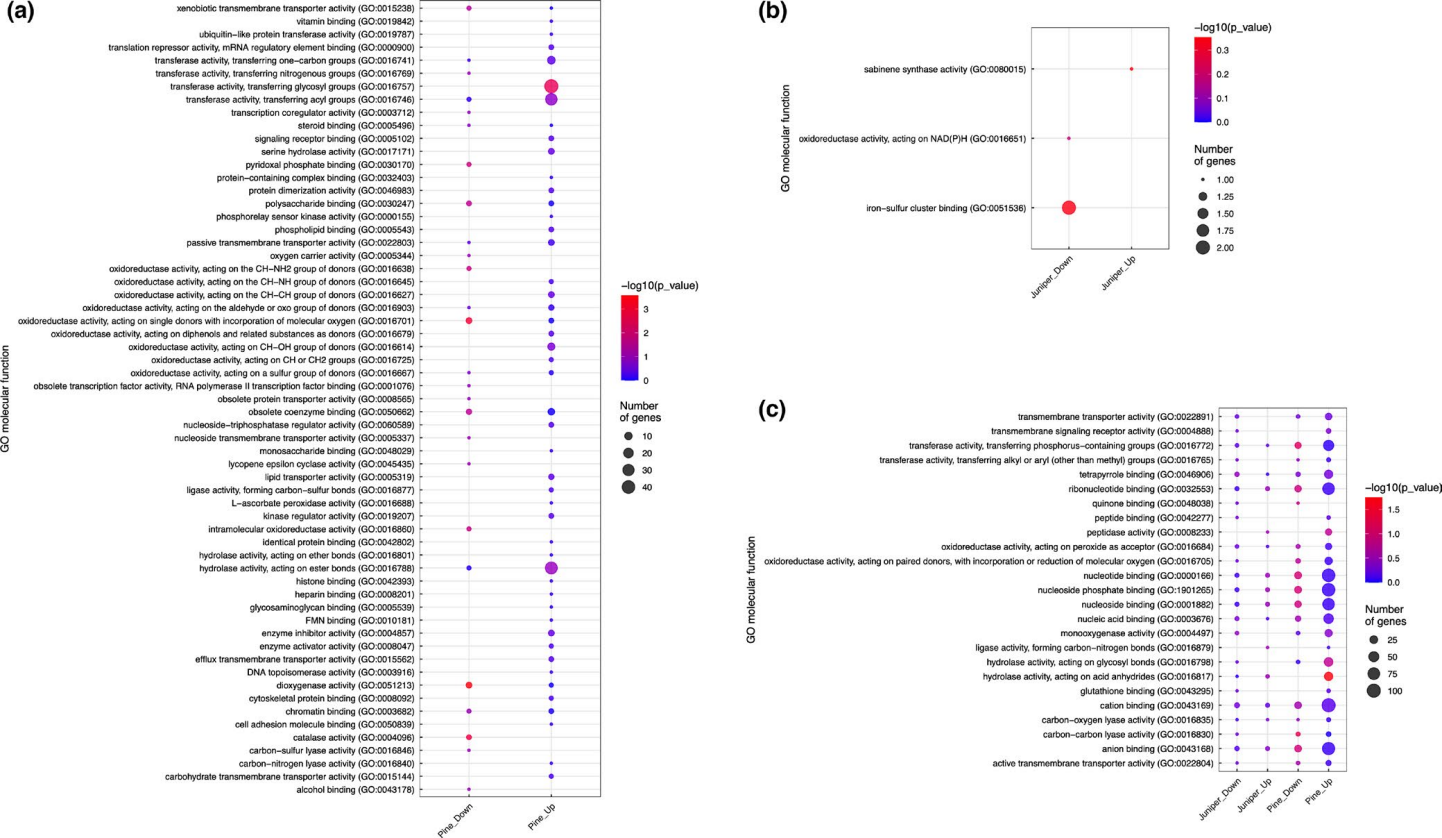
Molecular function gene ontology (GO) enrichment analysis. Enriched GO terms of up‐ (juvenile) and down‐ (adult) regulated genes in *J. flaccida* and *P. cembroides* were ranked by ‐log10(*p*‐value) and number of genes utilizing GOseq. Sixty‐one enriched terms were unique to *P. cembroides* (a), three were unique to *J. flaccida* (b), and 25 were shared between both species by at least one condition (c)

Analysis of enriched *molecular function* Gene Ontology (GO) terms from up‐ and down‐regulated transcripts revealed 61 terms unique to *P. cembroides*, three unique to *J. flaccida*, and 25 shared between both species. Of the 61 terms specific to *P. cembroides*, 13 were shared between both conditions, 35 were uniquely up‐regulated, and 13 were uniquely down‐regulated (Figure 4a). Of those shared, hydrolase activity, acting on glycosyl bonds (GO:0016798), and transferase activity, transferring acyl groups (GO:0016746), were the most enriched. Meanwhile, transferase activity, transferring glycosyl groups (GO:0016757), and dioxygenase activity (GO:0051213) were most significantly enriched in the juvenile and adult condition, respectively. By comparison, none of the terms unique to *J. flaccida* were shared between the juvenile and adult conditions (Figure 4b). Iron–sulfur cluster binding (GO:0051536) and oxidoreductase activity, acting on NAD(P)H (nicotinamide adenine dinucleotide phosphate) (GO:0016651), were enriched in the adult juniper, while sabinene synthase activity (GO:0080015) was uniquely expressed in the juvenile. Of the 25 terms shared between both species (by at least one condition), 11 terms were shared by the adult and juvenile conditions in both *J. flaccida* and *P. cembroides* (Figure 4c). Anion binding (GO:0043168), cation binding (GO:0043169), and nucleotide binding (GO:0000166) were most significant. Juvenile *J. flaccida* had two overlapping terms with juvenile *P. cembroides*, ligase activity, forming carbon‐nitrogen bonds (GO:0016879), and peptidase activity (GO:0008233). It also had one term, hydrolase activity, acting on acid anhydrides (GO:0016817), shared with juvenile *P. cembroides* and adult *J. flaccida*. By comparison, adult *J. flaccida* shared a single term, quinone binding (GO:0048038), with adult *P. cembroides*, three terms with juvenile *P. cembroides*, and seven with both juvenile and adult *P. cembroides*. Those shared uniquely with juvenile *P. cembroides* had very few genes, but transmembrane signaling receptor activity (GO:0004888) was the most enriched. Meanwhile, monooxygenase activity (GO:0004497) and carbon‐carbon lyase activity (GO:0016830) made up the most significant terms adult *J. flaccida* shared between both *P. cembroides* conditions.

### 2.5 Gene family analysis

**FIGURE 5:**
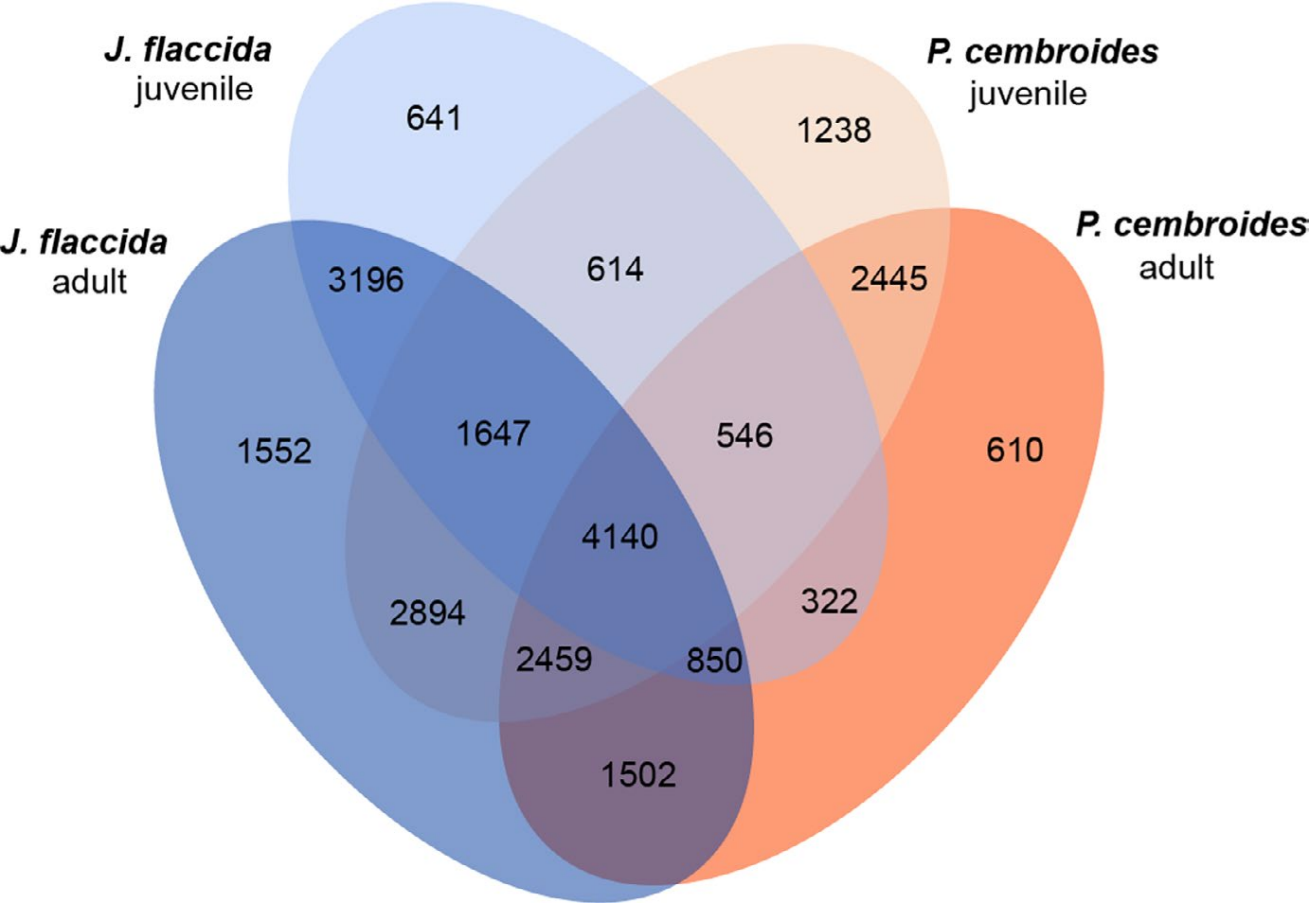
Gene family comparison of juvenile and adult conditions in *J. flaccida* and *P. cembroides*. Venn diagram depicting the unique and shared gene families in the proteins (derived from the assembled transcriptomes) of *J. flaccida* (juvenile: light blue; adult: dark blue) and *P. cembroides* (juvenile: light orange; adult: dark orange)

The four annotated and translated transcriptomes (pine and juniper with juvenile and adult stages) were compared. A total of 155,102 genes were assigned to 34,448 different orthologous gene families, with a surprising few, 4140 (12%), shared among all four (Figure 5). This protein‐level comparison allows a general examination of shared and unique gene families that is robust to some of the fragmentation and errors resulting from de novo transcriptome assembly. As expected, these shared families correspond to activities related to constitutive metabolic components, such as photosynthesis elements, transcription factors, and regulatory factors of secondary metabolites. The most abundant orthogroup described the regulation of mitotic recombination (GO:0000019), and included 49 genes, and the regulation of DNA recombination (GO:0000016), which included 51 genes.

Comparing juvenile and adult pines exclusively, 18,638 genes (29.2% of the combined gene set) were grouped into 4293 orthologous gene families (family must have at least two genes). Of these, 1238 gene families were unique to the juvenile condition, 610 to the adult one, and 2445 were shared by both. The juvenile‐specific orthologous gene families saw increased activity of the G protein‐coupled amine receptor (GO:0008227) and the short‐chain fatty acid import transport (GO:0015913); meanwhile, adult‐specific families were enriched for amino acid transmembrane transporters (GO:0015359) and ABC‐type nickel transporters (GO:0015413).

In contrast, adult and juvenile junipers had 22,177 (31.9% of the combined gene set) genes grouped into 5389 orthologous gene families. A total of 641 were unique to the juvenile condition, 1552 to the adults, and 3196 shared. Among juveniles, peroxisomal membrane transport (GO:0015919) and formate oxidation activity (GO:0015413) were most abundant. The adult juniper families were enriched for isoprenoid metabolic process (GO:0016096) and nonspecific RNA polymerase II transcription factor activity (GO:0016252). Orthogoups related directly to meristem growth and morphogenesis were not observed in the same stage for both species. Gene families related to meristem initiation activities (GO: 0010014); for example, only had one or two genes present in all transcriptomes, with the exception of the juvenile juniper.

### 2.6 Network analysis

**FIGURE 6:**
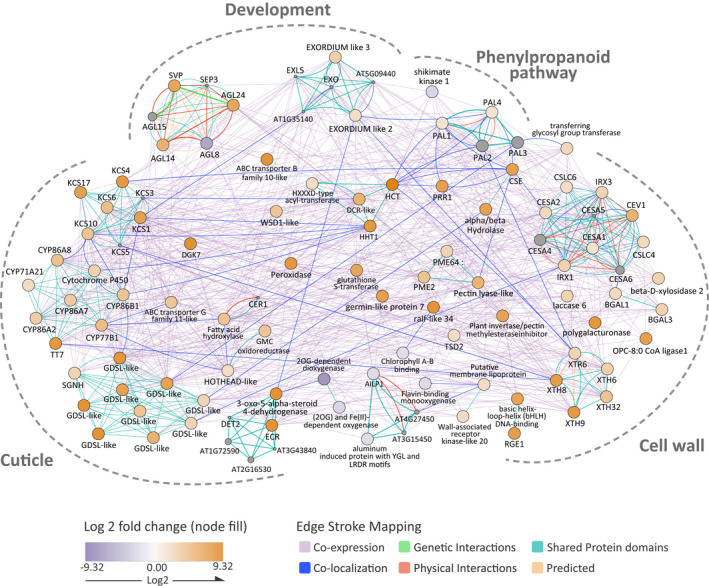
*P. cembroides* gene interaction network. Juvenile (orange circle) versus adult (purple circle) differentially expressed genes (via reference organism *Arabidopsis thaliana*) associated with cuticle and cell wall synthesis, development, and the phenylpropanoid pathway (grouped under dashed lines) collectively run through GeneMANIA. Related genes (gray circle) and co‐expression interactions (purple), genetic interactions (green), physical interactions (pink), predicted interactions (orange), shared protein domains (teal), and co‐localization interactions (blue) are represented. The fold change significance of the eight down‐regulated and 78 up‐regulated genes is depicted by the increased color saturation from the midpoint of zero to −9.32 and 9.32

The networks of *P. cembroides* differentially expressed genes were divided into four biological functional groups: cuticle, development, phenylpropanoid pathway, and cell wall (Figure 6). Up‐regulated *P. cembroides* genes grouped by cuticle formation included β‐ketoacyl‐CoA synthase (KCS), cytochrome P450 (CYP), and GDSL esterases and lipases, whereas down‐regulated *P. cembroides* saw 2‐oxoglutarate (2OG)‐dependent oxygenase functionality. Up‐regulated genes involved in leaf development included MADS‐box (AGL, SEP3, SVP) associated genes and EXORDIUM‐like (EXL, AT5G09440, AT1G35140) genes, whereas down‐regulated *P. cembroides* genes included Agamous‐like MADS‐box protein AGL8 and shikimate kinase 1. Finally, regarding cell wall biogenesis, up‐regulated *P. cembroides* included phenylalanine ammonia‐lyase (PAL), cellulose synthase (CESA [cellulose synthase A], IRX [irregular xylem], CSLC [cellulose synthase‐like C]), and xyloglucan endo‐transglucosylase/hydrolase (XTH). Down‐regulated *P. cembroides* genes of the cell wall uniquely included chlorophyll A‐B binding, flavin‐binding monooxygenase, and aluminum‐induced protein with YGL and LRDR motifs AILP1.

**FIGURE 7:**
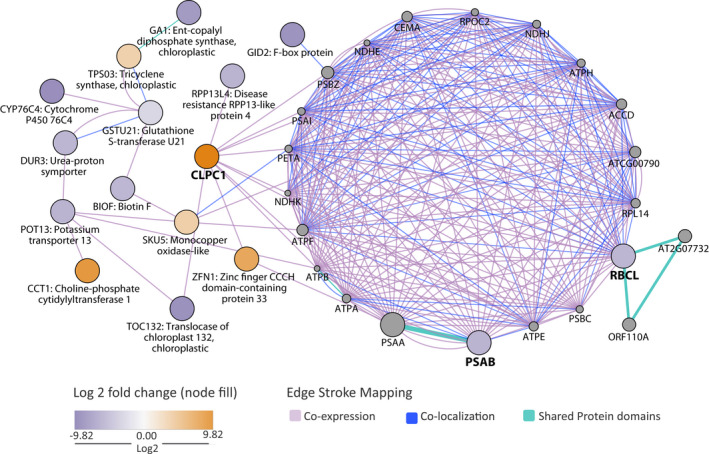
*J. flaccida* gene interaction network. Juvenile (orange circle) versus adult (purple circle) differentially expressed genes associated with photosynthetic functionality viewed with GeneMANIA‐related genes (gray circle), yielding co‐expression interactions (purple), co‐localization interactions (blue), and shared protein domains (teal). The fold change significance of 11 down‐regulated and five up‐regulated genes is depicted by the increased color saturation from the midpoint of zero to −9.82 and 9.82

As shown by the clustering of resultant genes (e.g., PSAA, PSBZ), *J. flaccida* enriched genes were primarily characterized by photosynthetic functionality (Figure 7). Uniquely, down‐regulated *J. flaccida* saw ribulose bisphosphate carboxylase (RBCL) activity, as well as activity from photosystem I P700 chlorophyll a apoprotein A2 (PSAB). Comparatively, up‐regulated *J. flaccida* saw monocopper oxidase‐like (SKU5) activity, as well as zinc finger CCCH domain‐containing protein (ZFN1) and ATP‐dependent Clp protease activity of subunit ClpC homolog 1 (CLPC1).

## 3 DISCUSSION

Heteroblasty in junipers and pines involves changes in the morphology and anatomy of the leaves, as well as the shoot structure and growth. Comparatively, the changes between the juvenile and the adult phase in both genera are of a different nature. On one hand, *J. flaccida* shows drastic changes in leaf size and the internodal distance (Figure 1a–c). These changes imply a reduction in the number of stomata and a marked reduction in their exposed surface area in adult leaves (Figure 2a–h). These characteristics of the adult phase decrease the evapotranspiration but at the cost of reduced CO_2_ uptake. This has been observed through ecophysiological analyses in *J. occidentalis*, for which a greater assimilation of CO_2_, leaf conductance, and transpiration in juvenile leaves have been reported (Miller et al., 1995). In addition, these modifications are accompanied by changes in the internal anatomy of the adult leaves, which do not present conductive tissues and show the resin duct occupies a greater internal area of the leaf (Figure 2h). On the other hand, juvenile and adult leaves of *P. cembroides* do not show such striking morpho‐anatomical differences since they have similar exposed surface area and stomatal distribution. However, higher rates of CO_2_ assimilation (Wright, 1970) and higher biomass allocation in juvenile leaves have been observed in varying levels in several species of pines (Climent et al., 2011).

### 3.1 Transcriptome assembly

High‐throughput mRNA sequencing is especially suitable for profiling in non‐model organisms that lack genomic sequence data (Eldem et al., 2017). Here, we used RNA‐Seq to examine differences between juvenile and adult leaves of *P. cembroides* and *J. flaccida* transcriptomes from de novo assemblies. The three individual trees sampled as juvenile and adult stages were assessed on the landscape. As such, informatic processes applied careful filters for contaminants, explained by the nature of the sampling condition in their natural distribution, given the ecology interactions with symbionts and parasitic organisms. Here, we removed 3.26% of the gene space in *P. cembroides* and 3.11% in *J. flaccida*. *Cryptotermes secundus*, a wood ingesting insect, was detected in the juvenile stage of both species, and *Bipolaris oryzae* was detected in both stages. These two de novo assembled needle transcriptomes represent the very first effort to examine the molecular aspects of heteroblasty in conifers.

### 3.2 Genes implicated in cuticle biosynthesis and deposition are up‐regulated in juvenile leaves of *P. cembroides*

Several of the up‐regulated genes in juvenile leaves of *P. cembroides* have previously been associated with cuticle synthesis and deposition in *Arabidopsis* and other angiosperms (Yeats & Rose, 2013). Plant cuticles are generally constituted by a wax fraction compound by organic solvent‐soluble lipids, and an insoluble cutin fraction (Yeats & Rose, 2013). Particularly in conifers, an important quantity of lignin‐like polymers is present as well in the insoluble fraction of the cuticle (Kögel‐Knabner et al., 1994; Reina et al., 2001). Wax and cutin are products of the hydroxy fatty acid biosynthetic pathway for which different genes and gene families have been identified (Yeats & Rose, 2013). Different members of the 3‐ketoacyl‐CoA synthase gene family (KCS), which play an important role in wax biosynthesis, were found up‐regulated in juvenile leaves with many co‐expression and co‐localization interactions in the network (Figure 6), including transcripts with similarity to the *KCS1*, and *KCS6* (*CER6*) genes of *Arabidopsis*, for which mutant phenotypes with marked wax reduction have been described (Todd et al., 1999; Yeats & Rose, 2013). Wax lacking phenotypes (*cer*) have been associated as well with other gene families, such as the gene *ECERIFERUM1* (*CER1*) that codes a fatty acid hydroxylase protein involved in alkane biosynthesis, and *CER10*, a very‐long‐chain enoyl‐CoA reductase (ECR; Figure 6; Yeats & Rose, 2013; Zheng et al., 2005). Although in our analysis we did not find transcripts similar to *CER1*, we found a transcript with a shared domain and thus with probable physical interaction with this gene (Figure 6). Among the differentially expressed genes of the cytochrome P450 protein family are the orthologs of CYP86 and CYP77 subfamilies (Figure 6), involved in polyester monomer processing for cutin biosynthesis (Li‐Beisson et al., 2009; Yeats & Rose, 2013).

Once the wax and cutin have been synthesized, they are transported throughout the cell wall, probably by means of the activity of two ATP‐binding cassette (ABC) transporters: ABCG12 (*CER5*) and ABCG11 proteins, which are required for wax export and cutin accumulation in angiosperms (Hwang et al., 2016; Yeats & Rose, 2013). Although we did not detect a transcript similar to ABCG12 in our analyses, the up‐regulation of an ABCG11 *P. cembroides* ortholog indicates a common mechanism of cuticle deposition in seed plants. In addition to the genes directly identified in the synthesis and transport of wax and cutin, other genes related to the modification and deposition of the cuticle were identified. For example, several transcripts up‐regulated in juvenile leaves belong to the GDSL lipase family (GDSL‐Like; Figure 6), which have been involved in wax synthesis and cutin deposition in different angiosperm species (Ma et al., 2018), and *HOTHEAD* (*HTH*), a glucose (Glc)‐methanol‐choline (GMC) oxidoreductase required for proper cuticle organization (Krolikowski et al., 2003). Furthermore, a set of transcripts for acyltransferases implicated in cuticle and cell wall modification were also up‐regulated. The O‐acyltransferase WSD1 gene plays a key role in wax ester synthesis in *Arabidopsis* (Li et al., 2008) and the BAHD‐acyltransferase proteins modify a variety of plant metabolites, including shikimate‐phenylpropanoid derivatives (Gou et al., 2009), and participate both in cuticle and cell wall formation. Specifically, similar transcripts to BAHD/HXXXD and DCR‐like BAHD‐acyltransferase genes, implied in the formation of cutin polyesters (Cheng et al., 2013; Panikashvili et al., 2009), were identified.

### 3.3 Genes involved in the synthesis of the primary and secondary walls are up‐regulated in juvenile leaves of *P. cembroides*

Genes related to the formation of primary and secondary cell walls were also found to be differentially expressed in pine juvenile and adult plants. Primary and secondary plant cell walls are mainly composed by cellulose and hemicellulose polysaccharides, which are assembled by the activity of cellulose synthase proteins (CESA) (Kumar & Turner, 2015) and plant glycosyltransferase proteins encoded by xyloglucan endo‐transglycosylase/hydrolase (XTH) genes (Eklöf & Brumer, 2010), respectively. Specifically, orthologs of CESA 4, 7, and 8 genes, associated with secondary cell walls in *P. taeda* (Nairn & Haselkorn, 2005), were retrieved in the interaction network (CSLC4, IRX3, IRX1; Figure 6), as well as transcripts with similitude to CESA 1 and 6, associated with primary wall synthesis (Kumar & Turner, 2015). Differences in the composition of the primary and secondary walls determine their functional differences. Primary walls are synthesized during cell growth and contain a high proportion of pectins, so they are more extensible allowing greater water uptake and an increase in size, while the secondary walls, deposited once the cell finished growing, have a higher lignin content, with greater rigidity and support (Cosgrove & Jarvis, 2012). In juvenile leaves of *P. cembroides*, transcripts identified as pectin methylesterases (PMEs) and several genes of the phenylopropanoid pathway, in which lignin is synthesized, were found to be up‐regulated (Figure 6). Particularly, we found orthologous genes to phenylalanine ammonia‐lyase (PAL), quinate/shikimate *p*‐hydroxycinnamoyltransferase (HCT), and caffeoyl shikimate esterase (CSE). Participation of these genes in the composition and quantity of lignin in plant cells has been tested in different species (Xie et al., 2018) and specifically the participation of the HCT gene in lignin biosynthesis in *P. radiata* (Wagner et al., 2007). Not all the phenylpropanoid metabolism genes detected in the differential expression analysis were recovered in the interaction network, but various genes of this metabolic pathway were up‐regulated in the juvenile phase. Although it has been highlighted that both the genes involved in the processing of cellulose and in lignin are regulated by different MYB‐type transcription factors (Nakano et al., 2015; Xie et al., 2018), in our analysis we did not detect any MYB gene with differential expression. However, MYB transcription factors were annotated in the adult reference transcriptomes (adult juniper: 360; adult pine: 289 sequences). A more detailed analysis revealed that many of these genes are expressed in both juvenile and adult tissues.

### 3.4 Relationship of the biological function of the DE genes with the differences of the juvenile and adult leaves of *P. cembroides*

Analysis on the timing of developmental transition from juvenile to adult phase in several pine species growing in common environments has shown variation in the ontogenetic patterns for different species (Climent et al., 2011; Lester, 1968; Pardos et al., 2009). More detailed studies have postulated that pines from drier climates tend to have longer juvenile phases (Climent et al., 2011; Pardos et al., 2009), which is also related to greater differences in the needle mass per unit (leaf mass per unit area (LMA)) and the rate of cuticular transpiration (RCT) between juvenile and adult leaves (Pardos et al., 2009). Given that juvenile leaves showed higher RCT and lower LMA than adult ones in pine species from arid or semi‐arid environments, the delay in transition to adult phase is interpreted as an adaptation to water stress (Pardos et al., 2009); even though juvenile leaves tend to lose more water, they are less expensive in terms of LMA, which allows the plant to survive in drought conditions and allocate more resources to adult leaves. Although the time for the transition to the adult phase has not been documented in *P. cembroides*, from personal observations we can assure that in pinyon pines that have diversified into arid and semi‐arid environments of North America (Ortiz‐Medrano et al., 2016), it is common to find juvenile plants of more than 1 year. In juvenile shoots, leaves are continuously developing throughout the year, while in the adult phase needles are differentiated in the winter meristem and grow during spring. Therefore, the up‐regulation of genes involved in synthesis of the cell wall and wax elements of cuticle is consistent with the development of juvenile shoots, which have shown a higher allocation of biomass to leaves than to other parts of the plant in several pine species (Climent et al., 2011). Moreover, the glaucous appearance of juvenile coniferous leaves (Figure 1b,e) is attributed to the extensive coverage of wax crystals during this phase of development, which probably function as a photoprotection (Stabentheiner et al., 2004). However, the up‐regulation of genes involved in lignin synthesis (phenylpropanoid pathway) does not have a direct association with juvenile leaves’ traits. Lignin is deposited on the cell wall of different plant tissues, mainly in the tracheids of the xylem, being one of the main components of wood. In coniferous leaves, the lignin that is part of the cuticle reduces evapotranspiration and increases the rigidity of the needles (McAdam & Brodribb, 2014). On the one hand, high levels of RCT could indicate that the cuticle of juvenile leaves has a low content of lignin, since it has been proven that this component of the cuticle is the one that most reduces transpiration (Reina et al., 2001). On the other hand, the stiffness observed in adult leaves can be attributed to a higher lignin content in the needles than in juvenile leaves. Therefore, the characteristics of the juvenile leaves indicate lower lignin content than the adult needles, despite the up‐regulation of genes of phenylpropanoid metabolism, probably because the recently synthesized lignin is allocated to other tissues, such as the stem. Another possible explanation is that despite the lignin synthesis pathway being active, juvenile plants do not have enough resources to produce it, which is consistent with what was hypothesized by (Climent et al., 2006), since in extreme drought conditions juvenile plants of *P. canariensis* delay shift phase, probably because juvenile plants do not have enough resources, but they are capable of allocating more resources to the adult needles.

### 3.5 Developmental genes are differentially expressed in juvenile and adult plants of *P. cembroides*

Transcripts from two main developmental gene families (MADS‐box and EXORDIUM) were found to be differentially expressed in *P. cembroides* leaves. Specifically, we found in juvenile leaves three transcripts annotated as SHORT VEGETATIVE PHASE (SVP) and one as AGAMOUS‐like 14 (AGL14) up‐regulated (Figure 6), and one transcript with similitude to AGL8 down‐regulated (Figure 6). In *Arabidopsis*, SVP acts as flower repressor by direct repression of SOC1, which in turn acts as flowering promoter (Li et al., 2008). Our analysis indicates that in contrast to angiosperms, in pine juvenile leaves both types of genes are up‐regulated, even though interpretations of orthology and function conservation among angiosperms and gymnosperms MADS‐box genes are not straightforward. Gene phylogeny of MADS‐box genes has shown that independent duplications have occurred since the divergence of these two seed plant lineages (Gramzow et al., 2014). A phylogenetic analysis of our sequences indicates that the three SVP transcripts conform to a monophyletic clade along with other sequences from Pinaceae (File S4) that is sister to the SVP/AGL24 clade of angiosperms, confirmed by genes with diverse identified functions. For example, SVP duplicates have been related to growth‐dormancy cycles in *Malus domestica* and other species (Wu et al., 2017) and AGL24 promotes flowering in *Arabidopsis* (Gregis et al., 2008). Similarly, AGL14 belongs to a diversified gene clade of angiosperms (TM3/SOC1 clade) associated with meristem identity, flowering transition (Pérez‐Ruiz et al., 2015), and dormancy (Voogd et al., 2015). Phylogenetic analysis of these genes in gymnosperms indicates that this clade is the most diversified in conifers, showing multiple duplication events at least for the Pinaceae family (Gramzow et al., 2014), therefore it is likely that different copies have acquired diverse functions in conifers. The down‐regulated gene in juvenile leaves, a transcript identified as AGL8 (Picb_DE43051), belongs to an exclusive clade of conifers. Though this group of genes is closely related to the clade AGL8, the phylogeny indicates that it is not possible to identify the members of this clade with any orthologous copy of angiosperms (File S4). Although it is not possible to directly extrapolate the activities reported for genes in angiosperms, we can assert that genes involved in meristem identity and dormancy are related to the transition from the juvenile to the adult phase in pine trees, and that the expression of SVP genes in the juvenile phase is conserved among seed plants. Interestingly, transcripts with identity to EXORDIUM genes are up‐regulated in juvenile plants. These genes have been related to cell proliferation and expansion in angiosperms (Schröder et al., 2009), which is congruent with the constant activity of new leaf formation and indeterminate shoot growth in juvenile plants.

### 3.6 Evidence of functional and developmental differences in juvenile and adult leaves of *J. flaccida*

The DE analysis of juvenile and adult leaves of *J. flaccida*, in contrast to the samples of *P. cembroides*, detected fewer differentially expressed genes, and therefore it was not possible to find strong evidence of changes in specific active functions. However, DE transcripts allow some inferences to be made regarding the functional and developmental differences between juvenile and adult leaves in this species. Some DE genes are related to photosynthesis (Figure 7) and plastid development and function. Physiological differences between juvenile and adult leaves have been documented for *J. occidentalis* (Miller et al., 1995), showing that juvenile leaves have higher levels of CO_2_ assimilation, faster growth rates, and higher conductance. CO_2_ capture and water exchange are related to the disposition and number of stomata on leaves, since juniper juvenile plants bear longer open leaves (Figure 1b) with numerous stomata on the adaxial side (Figure 2), whereas in adults, adaxial side of scale‐like leaves is not exposed (Figure 1c), bearing lower stomata number (Figure 2). Therefore, adults can avoid desiccation by reducing gas exchange, and capturing less CO_2_. However, comparative studies on *Sabina vulgaris* (Cupressaceae) indicate that the low assimilation of CO_2_ in adults is compensated by a higher efficiency in carbon allocation (higher LMA), higher leaf area‐based photosynthetic rate, higher water‐use efficiency, and stronger tolerance of photoinhibition (Tanaka‐Oda et al., 2010).

Two important genes for photosystem I (PSI) function were found down‐regulated in juvenile leaves of *J. flaccida*: Photosystem I P700 chlorophyll a apoprotein A2 (psaB) and the ribulose bisphosphate carboxylase large chain (rbcL) (Figures 3 and 7). Down‐regulation of the rbcL gene could be related to the high levels of CO_2_ intake of juvenile leaves, given that the expression levels of rbcL decrease under high concentrations of CO_2_ (Cheng et al., 1998). Moreover, some genes related to growth were found differentially expressed, for example, it has been reported that the expression of the monocopper oxidase protein (SKU5; Figures 3 and 7) is related to growth processes in *Arabidopsis*, possibly participating in cell wall expansion (Sedbrook et al., 2002) and the gene for the clathrin heavy chain 1 (Figure 3), a type of protein critical for several developmental processes (Wang et al., 2013).

### 3.7 Heteroblasty examined in junipers and pines

The conifer MADS‐box gene DAL1 has been identified as the mediator of juvenile‐to‐adult‐transition in *Picea abies*, though this gene is first detectable in 3‐year‐old plants and increases its expression until the plant begins to produce cones (Carlsbecker et al., 2004); therefore, this gene is more likely associated with the transition from the vegetative to the reproductive adult phases than with juvenile to adult phase shift. The differential expression analysis presented here does not allow to recover expression differences when the genes are completely absent in one of the compared tissues; therefore, in accordance with what was previously reported for *Picea*, if the DAL1 orthologs are not expressed in the juvenile leaves, it will not appear differentially expressed in adults. Similarly in our analysis, no evidence of differential expression of *miR156* and *miR172* target genes (*SPL* and *AP2*, respectively) was found, which have been previously reported as responsible for the transition from juvenile to the adult phase in angiosperms (Huijser & Schmid, 2011), including tree species where the juvenile to adult shift usually is uncoupled with the vegetative to reproductive shift (Ostria‐Gallardo et al., 2016; Wang et al., 2013). Therefore, it would be convenient to carry out specific studies focused on the changes of the genes involved in this regulation pathway in conifers, in order to clarify whether this pattern is common to seed plants. Although our study yields new evidence on the differences between juvenile and adult leaves in conifers from two families, to determine whether environmental changes influence differential gene expression, broader studies are required including sampling at different times of the day and in different seasons. As well as comparative studies with a larger taxonomic breadth, and that include species evolving in other types of environments. In addition, a more detailed analysis of the biochemical components of the leaves is required, in order to determine the role played by lignin in the heteroblasty of conifers.

Developmental patterns during the juvenile phase are similar in both conifer species, even though adult leaf development shows contrasting differences. The adult plants of *J. flaccida* show a growth pattern similar to the juvenile phase, although with smaller leaves and shorter internodes (Figure 1a–c). In contrast, in the genus *Pinus* a drastic change in the developmental pattern is observed, since in the adult phase three different types of leaves are observed (heterophylly): cataphylls, sheath leaves, and photosynthetic needles (Figure 1). Cataphylls have a developmental pattern similar to the juvenile leaves (growing spirally in stems of indeterminate growth), while the sheath leaves and needles develop in dwarf shoots (fascicles; Figure 1d–g). Therefore, the phase shift in pines implies not only a change of leaf shape or size, but a change in the shoot structure, which probably explains why reversion to juvenile‐type development in branches of adult individuals is much more common in junipers than in pines. In accordance with this, through the comparative transcriptomic analyses, a greater number of genes with multiple interactions were detected to be differentially expressed in *P. cembroides* than in *J. flaccida* (Figures 6 and 7). This also indicates that the functional and developmental differences between the juvenile and adult stages of *J. flaccida* imply changes in the expression of a smaller number of genes.

The comparison of heteroblasty in the Cupressaceae and Pinaceae families shows similarities in the growth strategy, since in different species of these families it has been observed that juvenile plants allocate more resources to the continuous formation of leaves with lower mass per unit (LMA) than the adult ones (Climent et al., 2006; Miller et al., 1995; Tanaka‐Oda et al., 2010). Juvenile leaves of junipers and pines have been reported to have a higher photosynthetic rate and a higher leaf conductance (Miller et al., 1995; Wright, 1970), although variations in this pattern have been observed in pine species (Zobel et al., 2001). It is known that the timing to phase shift is variable within the genus *Pinus*, and particularly in species distributed in arid zones this shift can be delayed for more than a year. Assessment of time to the shift to adult phase, in relation to the environment in *P. canariensis*, indicates that transition is delayed especially under drought conditions, where juvenile leaves would be less functional, due to their high conductivity and evapotranspiration rate (Climent et al., 2006). The authors suggest that prolonged juvenile phases are probably not a survival strategy, but simply the result of an environmental restriction, since under drought conditions, the plant cannot accumulate enough resources for the construction of adult leaves. Interestingly, this hypothesis could explain the fact that lignin synthesis pathways are active in juvenile leaves of *P. cembroides*, during the phase when plant tissues show less lignification. Considering that juvenile leaves and cataphylls show similar developmental patterns, and that frequently the first plant dwarf shoots with adult needles are differentiated in the axils of juvenile leaves (Climent et al., 2006; Lester, 1968), it can be argued that the juvenile photosynthetic leaves become cataphylls in the adult stages, which is why the lignification pathways are active, but they do not have the resources to do so. Therefore, heteroblasty in pine trees is a complex process that involves the development of a second type of leaves (needles) and it is not simply a matter of modifying the size and shape of the leaf, but of a change in the nature of the development of the leaves.

## 4 MATERIALS AND METHODS

### 4.1 Field sampling

Recently formed shoots of juvenile and adult trees were collected in sympatric natural populations of *Pinus cembroides* Zucc. and *Juniperus flaccida* Schltdl. in the locality of Maguey Verde, Queretaro, Mexico (21°05′48.9″N, 99°41′36.6″W). The region has a semi‐arid environment, located in an altitudinal transition between a desert area and a coniferous forest (2280 mamsl). Both species have a wide and scattered distribution in the semi‐arid zones of Mexico, with some populations in the southern United States (Figure S1). Adult plant shoots were collected in trees with similar dimensions (to minimize an effect by age differences) from new branches exposed to sunlight. All samples were obtained at the same time of day. Collected shoots were placed in 50‐ml vials and transported in liquid nitrogen. Tissues were stored at −80°C until processing for nucleic acid isolation. A different set of samples were placed in vials with a 70% ethanol solution and preserved at 4°C for anatomical observations.

### 4.2 Sample processing

For anatomical observations, freehand sections of leaves preserved in ethanol 70% were stained with 1% safranin for 1 min, followed by a wash with isopropyl alcohol and a second staining with 1% fast green for 30 s, a second wash with 50% ethanol and a final wash with 100% isopropanol. The semi‐permanent slides were mounted with Canada balsam. Microscopic sections were observed under a Leica DM500 light microscope and images were captured with a Leica ICC50 HD system. Additionally, longer sections (0.5 cm) of the middle part of the leaf of pine samples or the entire leaf of juniper samples were mounted in electrically conductive, non‐porous carbon tape for scanning electron microscopy observations (SEM).

RNA extraction was performed from juvenile and adult leaves of both species. Because of morphological characteristics, entire juniper branches were used, while for pine it was possible to separate the leaves from the stem. Tissues were stored on liquid nitrogen after collection and preserved at −80°C until RNA extraction. The tissues were ground in liquid nitrogen and the total RNA extracted using a Quick‐RNA™ Miniprep Kit (Zymo Research Corporation) as per the manufacturer's protocol; samples were then treated with DNase1 (Zymo Research Corporation). Extractions were ribo‐depleted and Illumina TruSeq RNA libraries were constructed with the standard protocol (Illumina). Only three isolates were used to obtain the transcriptome per stage of development. Therefore, each species had six total samples—three from juvenile leaves and three from adult leaves (12 samples in total). All libraries were sequenced at the Genomics Sequencing Laboratory in the California Institute for Quantitative Biosciences (QB3) at the University of California, Berkeley on the Illumina HiSeq 2500 (100 bp PE).

### 4.3 Transcriptome assembly

sickle (v.1.33) trimming (minimum quality score 33 and minimum length 40 bp) was applied to the paired‐end libraries to remove poor quality bases and reads. kraken2 (v. 2.0.8‐beta) was then run using the standard plus protozoa and fungi (PlusPF) database to taxonomically classify sequencing reads (Wood et al., 2019). Because there was no reference for either species, the transcriptome was assembled de novo with trinity (v.2.6.6) (min contig length 300 bp) for each set of trimmed and paired libraries (Grabherr et al., 2011). The three samples per each age group and species were then concatenated into a single reference. transdecoder (v.5.3.0) was subsequently run on each reference to identify coding regions within the transcript sequences; long open reading frames were extracted first and then a Pfam search executed by hmmer (v.3.2.1) was conducted to identify open reading frames (ORFs) containing true domains (Finn et al., 2011). A nucleotide and a protein file were generated for each reference with the predicted ORF sequences. A DIAMOND search, implemented in entap (v.0.9‐beta) at 80/80 coverage, was performed against four databases: NCBI RefSeq (*complete*.*protein*.*faa*.*87)*, NCBI RefSeq *(plant*.*protein*.*faa*.*97)*, UniProt, and NCBI’s nr database (Buchfink et al., 2015; Hart et al., 2020). Each run generated a list of potential contaminants (bacteria, fungi, and insects) to be removed, with pinaceae (*P. cembroides*) and cupressaceae (*J. flaccida*) specified as the target taxa. Filtered transcriptomes were generated for each species. vsearch (v.2.4.3) (90% identity) was then run on the final reference sets for each species. The nucleotide coding sequences were clustered first, and the protein sequences were filtered to match (Rognes et al., 2016). EnTAP was subsequently run without the nr database at 50/50 coverage to detect any lingering contaminants, and once more with the addition of Gymno PLAZA to include a gymnosperm‐specific resource and TAIR for down‐stream analysis (Berardini et al., 2015; Proost et al., 2015). EnTAP annotation also provided gene family assignment via EggNOG and Gene Ontology terms. rnaquast (v.2.0.1) summarized each reference following Trinity, TransDecoder, and VSEARCH (Bushmanova et al., 2016). The output quantified the number of transcripts, approximating the length of each transcriptome. Likewise, busco (v.5.0.0) odb10 *viridiplantae* was run to assess completeness (Seppey et al., 2019). kallisto (v.0.44.0) was utilized to build an index per species, align the paired reads from each library, and generate raw and normalized counts (Bray et al., 2016). Samtools flagstat was next run against each individual BAM file to assess mapping rates (Li et al., 2009). As a comparison, both species were assembled without TransDecoder and mapping rates were quantified.

### 4.4 Differential expression annotation and gene ontology

Count files were imported to RStudio (Bioconductor) and DE genes were identified with deseq2 (v.1.26.0) (Love et al., 2014; Soneson et al., 2015). This analysis defined significant genes as those having a *p*‐adjusted value <.1 and a log2 fold change >1.5. Functional descriptions were extracted from the master EnTAP annotation. To perform the enrichment analysis, level 3 *molecular function* GO terms of differentially expressed genes were extracted from the EnTAP output, and effective lengths were derived from Kallisto's output tsv. GOseq was used to identify functionally enriched terms that were unique and shared between species, and ggplot2 produced a figure representing the number of genes and ‐log10(*p*‐value) of each term (Wickham, 2016; Young et al., 2010). The final reference sets for each age and species combination were run in orthofinder (v.2.4.0; Emms & Kelly, 2015) to infer orthologous gene families with sequence similarity searches performed by DIAMOND (Buchfink et al., 2015) (Table S6). The longest sequence from each putative gene family, derived from the EnTAP analysis, was used to assign an overall function to the group.

### 4.5 Network analysis

Using the putative *Arabidopsis* orthologs assigned from EnTAP, GeneMANIA was used in Cytoscape to identify network interactions (Shannon et al., 2003; Warde‐Farley et al., 2010). Each individual run had parameters set to automatic weighting and max resultant genes at 20. Differentially up‐ (juvenile) and down‐ (adult) regulated expressed genes of *P. cembroides* were processed together, generating a comparative network. A sub‐network exhibiting cell wall, cuticle, and developmental functionality was also processed. Differentially expressed genes of *J. flaccida* exhibiting photosynthetic functionality were processed as well. Networks were defined by color on an imported log2 fold change scale between −9.32 and 9.32 in *P. cembroides* and −9.82 and 9.82 in *J. flaccida* correlating to the query genes (juvenile vs. adult), where the spectrum of purple corresponds to down‐regulated genes (<0) and orange to up‐regulated genes (>0).

## CONFLICT OF INTEREST

The authors declare no conflict of interest.

## AUTHOR CONTRIBUTION

**Cynthia Webster:** Formal analysis (equal); Investigation (equal); Methodology (lead); Visualization (equal); Writing – original draft (equal); Writing – review & editing (equal). **Laura Figueroa‐Corona:** Formal analysis (equal); Investigation (equal); Visualization (equal); Writing – original draft (equal); Writing – review & editing (equal). **Iván David Méndez‐González:** Investigation (supporting); Writing – review & editing (equal). **Lluvia Álvarez‐Soto:** Investigation (supporting); Visualization (equal); Writing – review & editing (equal). **David Neale:** Funding acquisition (lead); Writing – review & editing (equal). **Juan P. Jaramillo‐Correa:** Funding acquisition (lead); Project administration (equal); Resources (equal); Writing – review & editing (equal). **Jill L. Wegrzyn:** Conceptualization (supporting); Funding acquisition (supporting); Project administration (equal); Resources (equal); Supervision (equal); Writing – original draft (equal); Writing – review & editing (equal). **Alejandra Vázquez‐Lobo:** Conceptualization (lead); Funding acquisition (supporting); Investigation (supporting); Project administration (equal); Supervision (equal); Visualization (equal); Writing – original draft (equal); Writing – review & editing (equal).

## Supporting information

Fig S1

Fig S2

Table S1

Table S2

Table S3

Table S4

Table S5

Table S6

File S1

File S2

File S3

File S4

## Data Availability

Details of full analysis, including intermediate files and supporting scripts, are publicly available at: https://gitlab.com/PlantGenomicsLab/rna‐seq‐comparison‐of‐young‐and‐adult‐leaves‐in‐juniper‐and‐pine and https://doi.org/10.5281/zenodo.5791571. The BioProject ID is PRJNA638086 and contains both the RNA‐Seq reads (Sequence Read Archive (SRA)) and the *de novo* assembled transcripts (Transcriptome Shotgun Assembly (TSA)). The National Center for Biotechnology Information (NCBI TSA) record for *P. cembroides* is GISD00000000 and for *J. flaccida*, GISB00000000.
