# Supplementary material for: Comparative analysis of differential gene expression indicates divergence in ontogenetic strategies of leaves in two conifer genera
Source: Ecol Evol. 2022 Feb 16;12(2):e8611. doi: 10.1002/ece3.8611 (PMC8848466; doi:10.1002/ece3.8611)
Supplement: Supplementary file 1 — Fig S1 [file ECE3-12-e8611-s009.docx]

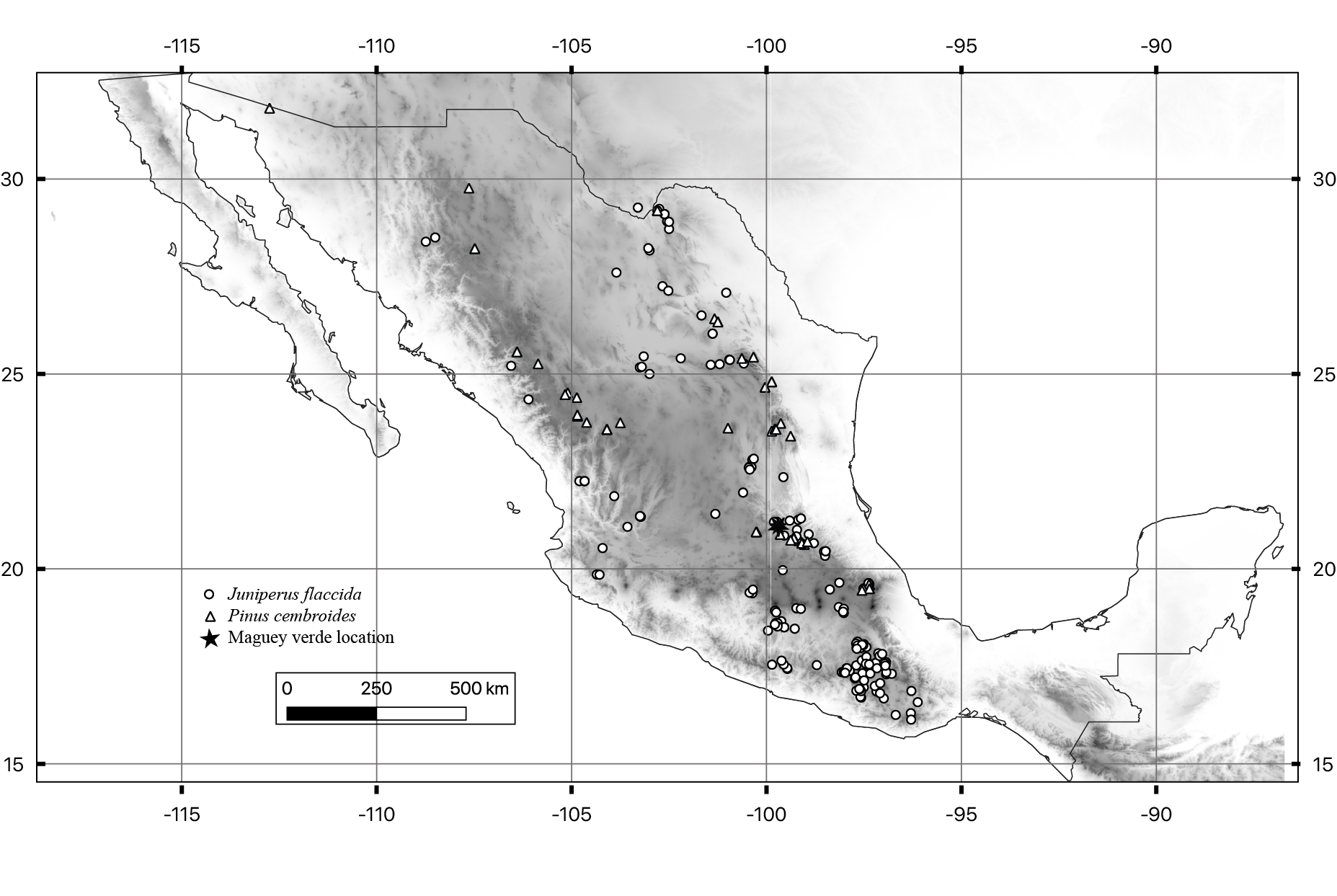


Figure S1**.** Site of sampling and native range of *J. flaccida* and *P. cembroides* throughout Mexico. The circles represent locations in Mexico where *J. flaccida* grows, whereas the triangles are indicative of *P. cembroides*. Maguey verde is the site of sampling for both species in the pinyon-juniper woodlands.
