# Supplementary material for: Comparative analysis of differential gene expression indicates divergence in ontogenetic strategies of leaves in two conifer genera
Source: Ecol Evol. 2022 Feb 16;12(2):e8611. doi: 10.1002/ece3.8611 (PMC8848466; doi:10.1002/ece3.8611)
Supplement: Supplementary file 2 — Fig S2 [file ECE3-12-e8611-s001.docx]

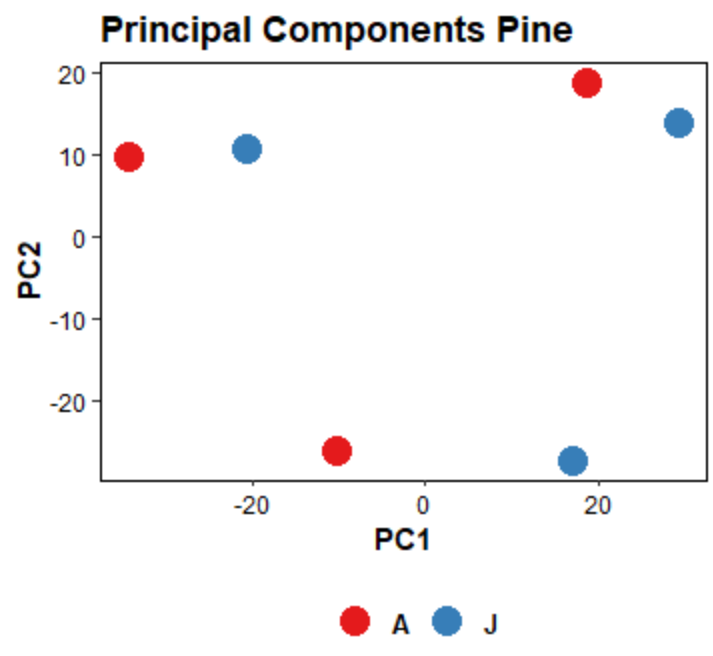

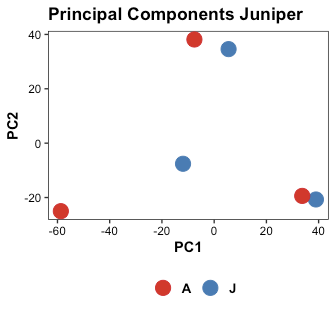


Figure S2. PCA plot of *P.cembroides* (left) and *J. flaccida* (right), illustrating adult (red) and juvenile (blue) samples, with the PC1 axis showing the greatest variance.
