# Supplementary material for: Comparative analysis of differential gene expression indicates divergence in ontogenetic strategies of leaves in two conifer genera
Source: Ecol Evol. 2022 Feb 16;12(2):e8611. doi: 10.1002/ece3.8611 (PMC8848466; doi:10.1002/ece3.8611)
Supplement: Supplementary file 3 — Table S1 [file ECE3-12-e8611-s011.docx]

Table S1. RNAQuast results following each software run in transcriptome assembly

| Sample | Transcripts | Transcripts > 500 bp | Transcripts > 1000 bp | Avg length of transcripts | Longest transcript | Total length | Transcript N50 |
| --- | --- | --- | --- | --- | --- | --- | --- |
| *De novo* Assemblies *-* Trinity (FASTA) | | |  |  |  |  |  |
| JF1A | 159283 | 66774 | 17534 | 588.661 | 5532 | 93763756 | 516 |
| JF2A | 78364 | 28349 | 5849 | 538.043 | 5511 | 42163238 | 2039 |
| JF3A | 84729 | 16336 | 1287 | 428.552 | 5096 | 36310760 | 348 |
| JF1J | 123905 | 35549 | 4790 | 478.675 | 4637 | 59310167 | 366 |
| JF2J | 20308 | 3907 | 311 | 427.049 | 3064 | 8672514 | 322 |
| JF3J | 110759 | 26496 | 2818 | 452.666 | 5150 | 50136847 | 1015 |
| PC1A | 68352 | 25958 | 6022 | 553.586 | 8114 | 37838696 | 1222 |
| PC2A | 63913 | 19694 | 2937 | 492.163 | 4912 | 31455590 | 433 |
| PC3A | 65228 | 23396 | 4272 | 525.753 | 5823 | 34293793 | 319 |
| PC1J | 77776 | 27419 | 4911 | 521.422 | 4997 | 40554142 | 471 |
| PC2J | 103756 | 35550 | 5859 | 513.244 | 10452 | 53252094 | 797 |
| PC3J | 82046 | 27185 | 4751 | 509.079 | 6502 | 41767897 | 1112 |
| Frame Selected - TransDecoder (CDS) | | |  |  |  |  |  |
| Juniper Combined | 229054 | 78234 | 12796 | 511.222 | 4881 | 117097365 | 717 |
| Pine Combined | 231054 | 82265 | 11970 | 511.606 | 6615 | 118208592 | 759 |
| Final Clustered Transcriptome - VSEARCH (CDS) | | | |  |  |  |  |
| Juniper Combined | 69448 | 21167 | 4247 | 502.07 | 4881 | 34867758 | 492 |
| Pine Combined | 63741 | 19344 | 3178 | 491.217 | 6615 | 31310688 | 1089 |
|  |  |  |  |  |  |  |  |
