# Supplementary material for: Comparative analysis of differential gene expression indicates divergence in ontogenetic strategies of leaves in two conifer genera
Source: Ecol Evol. 2022 Feb 16;12(2):e8611. doi: 10.1002/ece3.8611 (PMC8848466; doi:10.1002/ece3.8611)
Supplement: Supplementary file 4 — Table S2 [file ECE3-12-e8611-s006.docx]

Table S2. Busco completeness and EnTAP statistics per each species’ full transcriptome

|  | **Juniper Combined** | **Pine Combined** | |
| --- | --- | --- | --- |
| **BUSCO (PEP)** | | |  |
| Complete BUSCOs (C) | 154 (36.3%) | | 128 (30.1%) |
| Complete and single-copy BUSCOs (S) | 141 (33.2%) | | 108 (25.4%) |
| Complete and duplicated BUSCOs (D) | 13 (3.1%) | | 20 (4.7%) |
| Fragmented BUSCOs (F) | 191 (44.9%) | | 188 (44.2%) |
| Missing BUSCOs (M) | 80 (18.8%) | | 109 (25.7%) |
| **EnTAP (PEP)** | | |  |
| Total Sequences: | 69448 | | 63741 |
| Total unique transcripts w/ alignment | 24172 | | 23247 |
| Total unique sequences annotated (gene family assignment): | 22432 | | 22885 |
| Total unique sequences annotated (gene family and/or SS): | 46604 | | 46132 |
|  |  | |  |
