# Supplementary material for: Comparative analysis of differential gene expression indicates divergence in ontogenetic strategies of leaves in two conifer genera
Source: Ecol Evol. 2022 Feb 16;12(2):e8611. doi: 10.1002/ece3.8611 (PMC8848466; doi:10.1002/ece3.8611)
Supplement: Supplementary file 5 — Table S3 [file ECE3-12-e8611-s003.docx]

Table S3: Sample mapping rates including frame selection

| **Sample** | **Total Reads** | **Pseudoaligned** | **Mapping Rate** |
| --- | --- | --- | --- |
| PC3J | 25,090,745 | 12,015,702 | 47.9% |
| PC3A | 23,749,158 | 12,168,255 | 51.2% |
| PC2J | 25,922,170 | 13,065,050 | 50.4% |
| PC2A | 23,085,805 | 12,150,217 | 52.6% |
| PC1J | 21,015,370 | 11,209,297 | 53.3% |
| PC1A | 18,627,701 | 10,092,287 | 54.2% |
| JF3J | 31,132,040 | 13,531,281 | 43.5% |
| JF3A | 31,881,672 | 12,745,544 | 40.0% |
| JF2J | 27,937,789 | 12,188,737 | 43.6% |
| JF2A | 28,067,322 | 6,958,292 | 24.8% |
| JF1J | 34,138,252 | 15,619,258 | 45.8% |
| JF1A | 29,855,922 | 16,793,141 | 56.3% |
