# Supplementary material for: Comparative analysis of differential gene expression indicates divergence in ontogenetic strategies of leaves in two conifer genera
Source: Ecol Evol. 2022 Feb 16;12(2):e8611. doi: 10.1002/ece3.8611 (PMC8848466; doi:10.1002/ece3.8611)
Supplement: Supplementary file 6 — Table S4 [file ECE3-12-e8611-s010.docx]

Table S4: Sample mapping rates without frame selection

| **Sample** | **Total Reads** | **Pseudoaligned** | **Mapping Rate** |
| --- | --- | --- | --- |
| PC3J | 25,090,745 | 16,259,580 | 64.8% |
| PC3A | 23,749,158 | 15,746,879 | 66.3% |
| PC2J | 25,922,170 | 17,016,495 | 65.6% |
| PC2A | 23,085,805 | 15,761,933 | 68.3% |
| PC1J | 21,015,370 | 14,442,321 | 68.7% |
| PC1A | 18,627,701 | 13,702,984 | 73.6% |
| JF3J | 31,132,040 | 19835200 | 63.7% |
| JF3A | 31,881,672 | 18,720,562 | 58.7% |
| JF2J | 27,937,789 | 17,573,964 | 62.9% |
| JF2A | 28,067,322 | 20,408,192 | 72.7% |
| JF1J | 34,138,252 | 23,163,481 | 67.9% |
| JF1A | 29,855,922 | 22,392,004 | 75.0% |
