# Supplementary material for: Comparative analysis of differential gene expression indicates divergence in ontogenetic strategies of leaves in two conifer genera
Source: Ecol Evol. 2022 Feb 16;12(2):e8611. doi: 10.1002/ece3.8611 (PMC8848466; doi:10.1002/ece3.8611)
Supplement: Supplementary file 7 — Table S5 [file ECE3-12-e8611-s004.docx]

Table S5: Taxonomic classification of trimmed raw reads

| **Sample** | **Trimmed reads** | **Classified reads** | **Chordate reads** | **Unclassified reads** | **Microbial reads** | **Bacterial reads** | **Viral reads** | **Fungal reads** | **Protozoan reads** |
| --- | --- | --- | --- | --- | --- | --- | --- | --- | --- |
| PC3J | 25,090,745 | 21.01% | 0.81% | 78.99% | 19.34% | 14.18% | 1.42% | 0.40% | 0.23% |
| PC3A | 23,749,158 | 22.86% | 0.74% | 77.14% | 21.27% | 16.45% | 1.62% | 0.41% | 0.21% |
| PC2J | 25,922,170 | 22.18% | 0.60% | 77.82% | 20.51% | 14.05% | 1.59% | 0.38% | 0.16% |
| PC2A | 23,085,805 | 33.93% | 0.49% | 66.07% | 32.15% | 25.18% | 1.86% | 0.45% | 0.31% |
| PC1J | 21,015,370 | 28.01% | 0.67% | 71.99% | 24.70% | 19.49% | 2.22% | 0.63% | 0.20% |
| PC1A | 18,627,701 | 31.11% | 0.78% | 68.89% | 29.17% | 21.06% | 2.98% | 0.27% | 0.16% |
| JF3J | 31,132,040 | 25.66% | 0.59% | 74.34% | 24.13% | 19.57% | 1.23% | 0.33% | 0.25% |
| JF3A | 31,881,672 | 22.88% | 0.46% | 77.12% | 19.45% | 16.34% | 1.19% | 0.41% | 0.24% |
| JF2J | 27,937,789 | 30.53% | 0.49% | 69.47% | 27.64% | 23% | 1.72% | 0.49% | 0.39% |
| JF2A | 28,067,322 | 69.63% | 0.51% | 30.37% | 68.10% | 46.02% | 0.53% | 7.07% | 2.58% |
| JF1J | 34,138,252 | 31.76% | 0.64% | 68.24% | 30.19% | 24.75% | 1.55% | 0.41% | 0.37% |
| JF1A | 29,855,922 | 27.08% | 0.96% | 72.92% | 25.03% | 19.25% | 1.48% | 0.48% | 0.13% |
