# Supplementary material for: Comparative analysis of differential gene expression indicates divergence in ontogenetic strategies of leaves in two conifer genera
Source: Ecol Evol. 2022 Feb 16;12(2):e8611. doi: 10.1002/ece3.8611 (PMC8848466; doi:10.1002/ece3.8611)
Supplement: Supplementary file 8 — Table S6 [file ECE3-12-e8611-s002.docx]

Table S6. OrthoFinder results between the whole proteome of both *J. flaccida* and *P. cembroides*

|  | AJ | AP | JJ | JP |
| --- | --- | --- | --- | --- |
| Number of genes | 44340 | 26348 | 25108 | 37393 |
| Number of genes in orthogroups | 35448 | 21996 | 20632 | 31062 |
| Number of unassigned genes | 8892 | 4352 | 4476 | 6331 |
| Percentage of genes in orthogroups | 79.9 | 83.5 | 82.2 | 83.1 |
| Percentage of unassigned genes | 20.1 | 16.5 | 17.8 | 16.9 |
| Number of orthogroups containing all species | 18240 | 12875 | 11956 | 15984 |
| Percentage of orthogroups containing all species | 74 | 52.2 | 48.5 | 64.8 |
| Number of species-specific orthogroups | 1552 | 610 | 641 | 1238 |
| Number of genes in species-specific orthogroups | 3981 | 1445 | 1547 | 3168 |
| Percentage of genes in species-specific orthogroups | 9 | 5.5 | 6.2 | 8.5 |
|  |  |  |  |  |
