# Supplementary material for: Comparative analysis of differential gene expression indicates divergence in ontogenetic strategies of leaves in two conifer genera
Source: Ecol Evol. 2022 Feb 16;12(2):e8611. doi: 10.1002/ece3.8611 (PMC8848466; doi:10.1002/ece3.8611)
Supplement: Supplementary file 12 — File S4 [file ECE3-12-e8611-s007.pdf]

**Supplementary Material 4. Phylogeny of MADS-box genes differentially expressed in juvenile and adult leaves of *P. cembroides*.**

Similar sequences of conifers and angiosperms were found through tblastx at the NCBI Genbank using as queries *P. cembroides* sequences and *Arabidopsis thaliana* selected genes. Sequences were translated to amino acids and aligned using MAFFT software online with a BLOSUM62 scoring matrix (https://mafft.cbrc.jp/alignment/software/). After alignment sequences were trimmed to exclude the highly variable C-terminus. Maximum Likelihood tree topology and support values were retrieved using RaxML Blackbox online server (https://raxml-ng.vital-it.ch/#/) with a BLOSUM62 substitution matrix and a Gamma parameter for among-site rate heterogeneity.

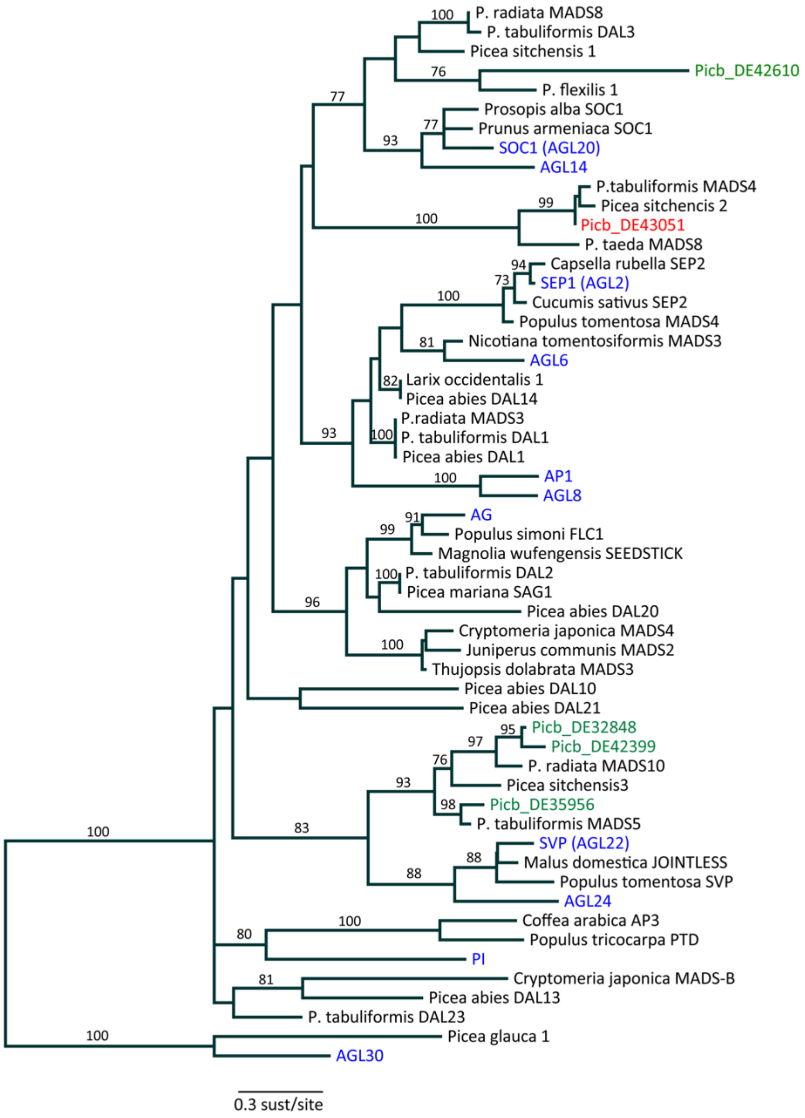


**Phylogeny of MADS-box genes differentially expressed in juvenile and adult leaves of *P. cembroides*.** *Arabidopsis* selected genes are denoted in blue. Numbers above branches indicate bootstrap values <70. Sequences upregulated in juvenile leaves of *P. cembroides* are denoted in green and downregulated in red.

**Table of names and accession numbers of sequences included in the MADS-box gene phylogeny.**

| Phylogeny label | Sequence name | Accession |
|---|---|---|
| AG | Arabidopsis thaliana K-box region and MADS-box transcription factor family protein (AG), partial mRNA | NM_118013 |
| AGL14 | Arabidopsis thaliana AGAMOUS-like 14 (AGL14), mRNA | NM_001340739 |
| AGL20 | Arabidopsis thaliana AGAMOUS-like 20 (AGL20), mRNA | NM_130128 |
| AGL24 | Arabidopsis thaliana AGAMOUS-like 24 (AGL24), mRNA | NM_118587 |
| AGL30 | Arabidopsis thaliana AGAMOUS-like 30 (AGL30), partial mRNA | NM_001335152 |
| AGL6 | Arabidopsis thaliana AGAMOUS-like 6 (AGL6), mRNA | NM_130127 |
| AGL8 | Arabidopsis thaliana AGAMOUS-like 8 (AGL8), mRNA | NM_125484 |
| AP1 | Arabidopsis thaliana K-box region and MADS-box transcription factor family protein (AP1), mRNA | NM_105581 |
| Capsella rubella SEP2 | PREDICTED: Capsella rubella developmental protein SEPALLATA 2 (LOC17892135), transcript variant X1, mRNA | XM_006298337 |
| Coffea arabica AP3 | Coffea arabica AP3 (AP3) mRNA, complete cds | KJ483229 |
| Cryptomeria Japonica MADS-B | Cryptomeria japonica mRNA for B-class MADS-box transcription factor, complete cds, clone: CMFL007_I11 | AB359036 |
| Cryptomeria Japonica MADS4 | Cryptomeria japonica isolate 1989-1004 MADS4 protein (MADS4) mRNA, complete cds | HM177453 |
| Cucumis sativus SEP2 | Cucumis sativus developmental protein SEPALLATA 2-like (CAGL2), mRNA | NM_001280738 |
| Juniperus communis MADS2 | Juniperus communis MADS2 protein (MADS2) mRNA, partial cds | HM177455 |
| Larix occidentalis 1 | Larix occidentalis isolate Cr15 MADS-box transcription factor gene, complete cds | KC214122 |
| Magnolia wufengensis SEEDSTICK | Magnolia wufengensis SEEDSTICK-like protein mRNA, complete cds | MK291518 |
| Malus domestica JOINTLESS | Malus x domestica JOINTLESS mRNA, complete cds | DQ402055 |
| Nicotiana tomentosiformis MADS3 | PREDICTED: Nicotiana tomentosiformis agamous-like MADS-box protein MADS3 (LOC104085632), mRNA | XM_009589724 |
| P. flexilis 1 | TSA: Pinus flexilis SS8075_c0_g1_i8, transcribed RNA sequence | GHWC01018447 |
| P. radiata MADS3 | Pinus radiata MADS-box protein (PrMADS3) mRNA, complete cds | U76726 |
| P. radiata MADS8 | Pinus radiata putative MADS box transcription factor PrMADS8 mRNA, complete cds | U90349 |

| | | |
|---|---|---|
| P. tabuliformis DAL1 | Pinus tabuliformis clone PtDAL1 DAL1 mRNA, complete cds | KJ711020 |
| P. tabuliformis DAL2 | Pinus tabuliformis clone PtDAL2 DAL2 mRNA, complete cds | KJ711027 |
| P. tabuliformis DAL23 | Pinus tabuliformis clone PtDAL23 DAL23 mRNA, complete cds | KJ711030 |
| P. tabuliformis DAL3 | Pinus tabuliformis clone PtDAL3 DAL3 mRNA, complete cds | KJ711031 |
| P. tabuliformis MADS4 | Pinus tabuliformis clone PtMADS4 MADS4 mRNA, complete cds | KJ711077 |
| P. tabuliformis MADS5 | Pinus tabuliformis clone PtMADS5 MADS5 mRNA, complete cds | KJ711078 |
| P. taeda MADS8 | Pinus taeda MADS-box protein 2 (Mads8) mRNA, complete cds | MH017255 |
| P.radiata MADS10 | Pinus radiata MADS-box transcription factor (MADS10) mRNA, complete cds | KM887510 |
| PI | Arabidopsis thaliana K-box region and MADS-box transcription factor family protein (PI), mRNA | NM_122031 |
| Picb_DE32848 | K-box region and MADS-box transcription factor family protein (SVP) | |
| Picb_DE35956 | K-box region and MADS-box transcription factor family protein (SVP) | |
| Picb_DE42399 | K-box region and MADS-box transcription factor family protein (SVP) | |
| Picb_DE42610 | agamous-like MADS-box protein (AGL14) | |
| Picb_DE43051 | AGAMOUS-like 8 | |
| Picea abies DAL1 | P. abies dal1 mRNA | X80902 |
| Picea abies DAL10 | Picea abies MADS-domain protein DAL10 (Dal10) mRNA, complete cds | AF064080 |
| Picea abies DAL13 | Picea abies clone DAL13-1 MADS-box transcription factor (DAL13) mRNA, complete cds | AF158543 |
| Picea abies DAL14 | Picea abies DAL14 protein (DAL14) mRNA, complete cds | KC347012 |
| Picea abies DAL20 | Picea abies MADS-domain transcription factor DAL20 (Dal20) mRNA, complete cds | KC305492 |
| Picea abies DAL21 | Picea abies DAL21 protein (DAL21) mRNA, complete cds | KC347016 |
| Picea glauca 1 | Picea glauca clone GQ04003_E19 mRNA sequence | BT118180 |
| Picea mariana SAG1 | Picea mariana AGAMOUS-like MADS-box transcriptional factor SAG1a mRNA, complete cds | U69482 |
| Picea sitchensis 1 | Picea sitchensis clone WS0453_A13 unknown mRNA | BT122582 |
| Picea sitchensis 2 | Picea sitchensis clone WS02765_F20 unknown mRNA | EF677316 |
| Picea sitchensis 3 | Picea sitchensis clone WS0461_J19 unknown mRNA | BT122820 |
| Populus simonii FLC1 | Populus simonii x Populus nigra isolate A flowering locus C (FLC1) mRNA, complete cds | JQ714386 |
| Populus tomentosa AGL24 | Populus tomentosa AGL24 gene, complete cds | KC297706 |

| | | |
|---|---|---|
| Populus tomentosa MADS4 | Populus tomentosa MADS4 mRNA, complete cds | DQ789398 |
| Populus tomentosa SVP | Populus tomentosa SVP gene, complete cds | KC297693 |
| Prosopis alba SOC1 | PREDICTED: Prosopis alba MADS-box protein SOC1 (LOC114726596), transcript variant X5, mRNA | XM_028913218 |
| Prunus armeniaca SOC1 | Prunus armeniaca SOC1 (SOC1) gene, complete cds | JX546224 |
| SEP1 (AGL2) | Arabidopsis thaliana K-box region and MADS-box transcription factor family protein (SEP1), mRNA | NM_001125758 |
| SVP (AGL22) | Arabidopsis thaliana K-box region and MADS-box transcription factor family protein (SVP), mRNA | NM_001335800 |
| Thujopsis dolabrata MADS3 | Thujopsis dolabrata isolate 1976-1028 MADS3 protein (MADS3) mRNA, complete cds | HM177454 |
